# GDPPH1 induces cell cycle arrest by downregulation of CDK4/Cyclin D1 via the m^6^A-METTL14-YTHDF2 axis, attenuating glioblastoma progression

**DOI:** 10.1038/s41419-026-09036-x

**Published:** 2026-07-03

**Authors:** Sijia Li, Ruijie Yuan, Jinxuan Su, Ruxi Chen, Xinyi Qu, Lele Zhao, Xiaofeng Zhou, Qiuming Zou, Zhijing Zhang, Siyu Yang, Jianqin Lai, Xiaosong Zhuang, Zisheng Li, Kai Cui, Shengwei Hu, Yongqi Liu, Lili Liu, Zepeng Luo, Zhongping Luo, Wen Li, Yu Yan, Yubo Zhang, Zhiming Zheng, Qi Qi

**Affiliations:** 1 State Key Laboratory of Bioactive Molecules and Drug ability Assessment, MOE Key Laboratory of Tumor Molecular Biology, Guangdong Province Key Laboratory of Pharmacodynamic Constituents of TCM and New Drugs Research, Department of Pharmacology, School of Medicine, Jinan University, Guangzhou, China; 2 Department of Dermatology, State Key Laboratory of Complex Severe and Rare Diseases, Peking Union Medical College Hospital, Chinese Academy of Medical Sciences and Peking Union Medical College, National Clinical Research Center for Dermatologic and Immunologic Diseases, Beijing, China; 3 State Key Laboratory of Cellular Stress Biology, Innovation Center for Cell Signaling Network, School of Life Sciences, Xiamen University, Xiamen, China; 4 Department of Physiology, Zunyi Medical University, Zunyi, Guizhou China; 5 Department of General Surgery, The First Affiliated Hospital of Jinan University, Guangzhou, China; 6 Department of Gastrointestinal Surgery, Guangzhou First People’s Hospital, Guangzhou, China; 7 State Key Laboratory of Cellular Stress Biology, Fujian Provincial Key Laboratory of Innovative Drug Target Research, School of Pharmaceutical Sciences, Faculty of Medicine and Life Sciences, Xiamen University, Xiamen, China; 8 Department of Respiratory and Critical Care, The First Hospital of China Medical University, Shenyang, China; 9 Department of Neurosurgery, Affiliated Hospital of Xiangnan University, Chenzhou, China; 10 Analytical and Testing Center, Jinan University, Guangzhou, China; 11 Functional Experimental Teaching Center, School of Medicine, Jinan University, Guangzhou, China; 12 Department of Neurosurgery, Shandong Provincial Hospital Affiliated to Shandong First Medical University, Jinan, China

**Keywords:** Pharmacodynamics, CNS cancer

## Abstract

Glioblastoma (GBM) is the most prevalent and aggressive primary brain tumor in adults, with current therapies failing to achieve significant clinical advancements. GDPPH1, a natural compound derived from *Garcinia mangostana L*., is known for its antioxidant properties; however, its anticancer effects and underlying mechanisms remain insufficiently understood. In this study, we demonstrate that GDPPH1 significantly inhibits GBM cell proliferation in a concentration-dependent manner by downregulating CDK4 and Cyclin D1 expression, inducing cell cycle arrest at G1/S checkpoint, and promoting apoptosis. Mechanistically, GDPPH1 reduces the m^6^A methylation of *CDK4* and *CCND1* through METTL14-YTHDF2-mediated transcript decay, resulting in decreased *CDK4*/*CCND1* expression and subsequent decreased RB phosphorylation levels. Further studies revealed that m^6^A-METTL14-YTHDF2 axis is responsible for GDPPH1-induced downregulation of CDK4 and Cyclin D1. Additionally, the in vivo anti-GBM efficacy of GDPPH1 was confirmed using both xenograft and orthotopic intracranial mouse models. Collectively, these findings provide robust evidence supporting GDPPH1 as a promising therapeutic candidate for GBM and offer novel insights into the role of m^6^A RNA methylation in cell cycle regulation.

## Introduction

Glioblastoma (GBM) is the most common malignant tumor of the central nervous system and is characterized by high aggressiveness, poor prognosis, and a low median survival rate [1]. Based on the differential expression of specific molecular markers, GBM is classified into proneural, proliferative, and mesenchymal subtypes. Among them, the mesenchymal subtype is associated with the poorest prognosis [2]. Currently, the alkylating agent temozolomide (TMZ), serves as the standard chemotherapeutic treatment for GBM patients [3]. However, similar to many other anticancer agents, TMZ’s clinical efficacy is hindered by low responsiveness and the development of drug resistance [4, 5]. Consequently, there is an urgent need to elucidate the molecular mechanisms underlying GBM progression, identify effective biomarkers, and develop novel therapeutic strategies to improve patient outcomes.

GDPPH1 is a natural compound extracted from *Garcinia mangostana L*. (mangosteen), a member of the *Clusiaceae* family [6]. The medical value of mangosteen is well-regarded; particularly due to xanthones, natural compounds isolated from its rind, such as α-Mangostin, γ-mangostin, guttiferones A [7]. These compounds have demonstrated significant antitumor efficacy, notably in inducing cell apoptosis [8], arresting the cell cycle [9], and suppressing metastasis [10, 11]. Given its biological activity, GDPPH1 represents a promising natural compound with substantial antitumor potential.

Post-transcriptional modifications of RNA play a crucial role in the regulation of RNA stability, transport, processing, and gene expression [12]. Among these, N^6^-methyladenosine (m^6^A) is the most prevalent and widely studied RNA modification, involving the methylation of adenosine (A) at the N^6^ position. This dynamic and reversible modification is mediated by RNA methyltransferases (“writers”), demethylases (“erasers”), and m^6^A-binding proteins (“readers”), collectively regulating RNA stability, splicing, degradation, and translation [13]. Emerging evidence has highlighted the pivotal role of m^6^A methylation in cancer development and progression [14, 15]. In GBM, several key components of the m^6^A regulatory machinery, including METTL14 and YTHDF family members [16, 17], are significantly upregulated compared with normal brain tissue, with YTHDF2 particularly enriched in high-grade GBM [18]. Functionally, YTHDF2 has been reported to promote glioma stem cell maintenance and tumorigenesis by stabilization of oncogenic transcripts such as MYC and VEGF [18], whereas depletion of METTL3 or METTL14 impairs GBM stem cell growth, self-renewal, and tumor formation [19]. In addition, elevated m^6^A levels are associated with increased invasiveness and chemotherapeutic resistance in GBM. GBM is also characterized by aggressive proliferation and profound dysregulation of the cell cycle, with 77% of GBM samples harboring RB pathway aberrations [20], representing a major barrier to effective therapy. Despite the growing recognition of m^6^A as a hallmark of tumor progression [21, 22], its functional contribution to cell cycle regulation in GBM remains poorly understood, highlighting m^6^A-mediated pathways as promising therapeutic targets in glioblastoma.

In the present study, we identified the anti-GBM effect and underlying mechanism of GDPPH1 both in vitro and in vivo. Our findings demonstrated that GDPPH1 diminishes the m^6^A methylation of *CDK4* and *CCND1* through METTL14-YTHDF2-mediated transcript decay, thereby downregulating the protein expression of CDK4 and Cyclin D1, blocking retinoblastoma protein (RB) signaling, suppressing GBM cell proliferation through inducing cell cycle arrest at G1/S phase, and promoting apoptosis. Moreover, the anti-GBM efficacy of GDPPH1 was confirmed in xenograft and orthotopic intracranial models in vivo. Hence, our study elucidates a novel epigenetic regulatory mechanism through which GDPPH1 exerts its anti-GBM effects, positioning GDPPH1 as a potential therapeutic candidate for GBM treatment.

## Materials and methods

### Chemical and reagents

The molecular structures and details of the nine candidate compounds, including GDPPH1, are summarized in Supplementary Table 1. GDPPH1 powder was extracted and purified independently by our research group. The chemical structure of the compound was elucidated by nuclear magnetic resonance (NMR) spectroscopy, and its purity was confirmed by high-performance liquid chromatography (HPLC) (Supplementary Fig. 1A-C, Supplementary Table 2). Stock solutions were prepared with dimethyl sulfoxide (DMSO) (Sigma Aldrich, St. Louis, MO, USA) and freshly diluted before experiments. Antibodies against CDK4 (#12790), Cyclin D1 (#55506), Retinoblastoma (#9309), Phospho-RB (Ser780) (#8180), β-tubulin (#2146), ERK1/2 (#4695), Phospho-ERK1/2 (Thr202/Tyr204) (#4377), AMPKα (#2532), and Phospho-AMPKα (Thr172) (#2535), p27 (#3686), Cyclin E1 (#20808), and β-actin (#3700) were purchased from Cell Signaling Technology. YTHDF2 (24744-1-AP), CDK2 (10122-1-AP), and E2F1 (66515-1-Ig) were purchased from Proteintech. METTL14 (F1391), YTHDF1 (F2505), and YTHDF3 (F2793) were purchased from Selleck. β-actin (T0022), Ki67 (#AF0198), and Cleaved Capsase-3 (Asp175) (AF7022) were purchased from Affinity. METTL3 (ab195352), ALKBH5 (16837-1-AP), and FTO (27226-1-AP) were purchased from Abcam. WTAP (TP74614) was purchased from Abmart. METTL14 (508530) used for IHC staining, was purchased from Zenbio. Actinomycin D (KM16333) was obtained from KKL Med Inc. Inhibitors, including Z-VAD-FMK (A834991) and Ferrostatin-1 (A228648) were purchased from Ambeed. Chloroquine (HY-17589A) and Necrostatin-1 (HY-15760) were purchased from MCE.

### Cell culture

Human glioblastoma cell lines U251, U118MG, U87MG, U87MG-luc, LN229, and LN18, as well as mouse brain endothelial cell line bEnd.3 were obtained from the American Type Culture Collection (ATCC). All cells were cultured in DMEM (Gibco, Invitrogen, Carlsbad, CA, USA) supplemented with 10% fetal bovine serum (Gibco, Invitrogen, Carlsbad, CA, USA) and 1% penicillin-streptomycin (MCE, China) at 37 °C in a humidified incubator with 5% CO_2_ (Thermo Fisher Scientific, USA).

### Animal study

Female BALB/c nude mice (5 – 6 weeks, 18–21 g) were purchased from Zhuhai BesTest Bio-Tech Co., Ltd, and were maintained in accordance with Jinan University’s Policy on the Care and Use of Laboratory Animals. All animal experiments were performed following institutional ethics and safety guidelines approved by Jinan University Institutional Animal Care and Use Committee. For the GBM xenograft subcutaneous model, U251 cells (2.0 × 10^6^) were suspended in serum-free substrate and engrafted into the right flank of nude mice. Once the tumor volumes reached 70 mm^3^, the mice were randomly divided into three groups receiving 2 mg/kg, 4 mg/kg GDPPH1/mouse/day, or 0.9% NaCl containing 0.5% DMSO (control) via peritoneal injection for 3 weeks. Tumor volumes were measured every two days using a vernier caliper through the skin and calculated as length (mm) × width^2^ (mm^2^) × 1/2. Mice were euthanized using CO_2_ at the end of study, and their hearts, livers, spleens, kidneys, and lungs were immediately fixed and paraffin-embedded for H&E staining.

For the GBM orthotopic model, mice were anesthetized and positioned on a stereotaxic frame. A Hamilton syringe with a 30-gauge needle was affixed to the frame, and 5 μl (2.5 × 10^5^ cells) of U87MG-luc cells were injected into the right brain (anteroposterior = 0.5 mm, middle = 2.0 mm, dorsoventral = 3.0 mm from bregma). Seven days later, mice were injected intraperitoneally with GDPPH1 (4 mg/kg/day) or vehicle for the duration of the survival period. Intracranial tumor growth was monitored on days 7, 14, and 28 using the IVIS Spectrum Live Imaging System (PerkinElmer, Branford, USA). Luciferase activity was quantified using Live Imaging Software (Xenogen). Mice were sacrificed when exhibiting signs of impaired activity or convulsion, and their brains were harvested and embedded in paraffin.

### Cell viability assay

Equal amount of cells (3.0 × 10^3^ cells/well) were seeded into 96-well plates and grown overnight. After treatment with GDPPH1 at various concentrations for 48 h, 3-(4, 5-Dimethylthiazol-2-yl)-2, 5-Diphenyltetrazolium Bromide (MTT) solution was added at a final concentration of 0.5 mg/mL, and the cells were incubated for an additional 4 h at 37 °C. The medium was carefully removed, and 150 µL of DMSO was added to dissolve the formazan crystals. Absorbance at 562 nm was measured using a continuous wavelength microplate reader (Varioskan LUX, Thermo Fisher Scientific). The absorbance values of each group were normalized to the control group, and cell proliferation and inhibition rate were calculated.

### Ethynyl deoxyuridine (EdU) assay

The Cell-Light^TM^ EdU staining kit (Ribobio, Guangzhou, China) was used for in vitro labelling of the nucleus in dividing cells. Cells were treated with 2 μM GDPPH1 and then incubated with 10 μM EdU for 16 h. Cells were fixed, and images were acquired using a confocal microscope (Zeiss).

### Calcein and propidium iodide (PI) staining

Cell density was adjusted to 1 × 10^5^ cells/ml, and cells were seeded in 6-well plates (2 mL/well). Cells were then incubated with different concentrations of GDPPH1. Viable and non-viable cells were detected using the Calcein/PI Cell Viability/Cytotoxicity Assay Kit (C2015M, Beyotime, China) according to the manufacturer’s instructions. Fluorescence images were captured using an inverted microscope for further analysis.

### Colony formation assay

Cells were seeded at 1000 cells/well in 6-well plates, with three replicates per group. After the cells were allowed to adhere, they were treated with different concentrations of GDPPH1, and the culture was continued for 14 days at 37 °C in a 5% CO_2_ incubator. Following treatment, the medium was aspirated, and the cells were washed twice with PBS. The cells were then fixed with methanol for 30 min and stained with 0.1% crystal violet (Beytime Biotechnology, Shanghai, China) at room temperature for 10 min. Colony quantification was performed using ImageJ software.

### RNA isolation and quantitative RT-PCR

RNA was extracted from cells using an RNA extraction kit (OMEGA Bio-Tek, USA). RNA was then reverse transcribed into cDNA using the RT Master Mix for qPCR II (MCE, USA). Real-time quantitative PCR was performed using ChamQ SYBR qPCR Master Mix (Vazyme, China). The primers for RT-PCR were synthesized by the BGI (BGI, Beijing, China). Primer sequences are listed in Supplementary Table 3. Relative expression levels were quantified using the 2^−ΔΔCt^ method, with normalization to reference genes.

### Western blotting

Cells or tissues were lysed using RIPA lysis buffer (Thermo Fisher Scientific), supplemented with PMSF and phosphatase inhibitor. Protein concentrations were measured using the BCA Protein Assay Kit (Thermo Fisher Scientific). A total of 20-50 μg of protein was loaded onto SDS-PAGE for separation and then transferred to 0.22 μm PVDF membranes (Millipore, USA). The membranes were blocked with 5% skim milk for 2 h and incubated with the appropriate primary antibody at 4 °C overnight. After washing, the membranes were incubated with HRP-conjugated secondary antibody (1:5000) at room temperature for 1 h. Protein expression was detected using Electrochemiluminescence (ECL) substrate reagents. Quantification was performed using ImageJ software v.1.8.0 (National Institutes of Health).

### Cell cycle analysis

Cell cycle analysis was performed using flow cytometry. After treatment with GDPPH1, cells were collected, fixed in cold 70% ethanol at 4 °C overnight, and stained with Propidium Iodide (Beyotime, China) at 37 °C for 30 min. The stained cells were analyzed using a flow cytometer (ACEA, USA).

### Analysis of mRNA m^6^A methylation

mRNA samples were diluted to concentrations of 400, 200, or 100 ng/μL and denatured by incubation at 70 °C for 5 min. Nitrocellulose membranes were prepared, with one membrane serving as an internal reference. Two microliters of each mRNA sample were applied to the membrane surface. After UV cross-linking, the membrane was incubated with 5% BSA to block non-specific binding. The membrane was then incubated with m^6^A antibody, and the subsequent experimental steps followed standard western blotting procedures. The internal reference was stained with 0.02% methylene blue and stained for 2 h. Signals were detected using an ECL kit.

### MeRIP-qPCR

The total mRNA was immunoprecipitated with pre-mixed m^6^A antibody magnetic beads, while a portion of the mRNA was retained as the input. The m^6^A immune magnetic beads were enriched using a magnetic rack after brief centrifugation. The mRNA-antibody complex was digested with proteinase, and the remaining mRNA was washed, dried, and resuspended in DEPC-treated water. *CDK4* and *CCND1* primers (Supplementary Table 4) were used for qPCR amplification of both input and MeRIP samples to calculate m^6^A modification.

### RNA immunoprecipitation (RIP) assay

RIP assays were performed using the Magna RIP RNA-Binding Protein Immunoprecipitation Kit (Millipore, 17-700) according to the manufacturer’s instructions. Cells were lysed with IP lysis buffer, and the lysates were divided into anti-YTHDF2, anti-IgG (control), and input samples. The lysates were incubated overnight at 4 °C with magnetic beads coated with 5 µg of specific antibodies. Following washing, the lysates were digested with Proteinase K, and the RNA bound to immunoprecipitated proteins was purified. Target RNA levels were quantified using qPCR.

### Isothermal Titration Calorimetry (ITC) assay

ITC measurements were performed using a MicroCal PEAQ-ITC instrument (Malvern). All experiments were conducted at 25 °C in a buffer comprising 600 mM NaCl and 5% DMSO. GDPPH1, loaded into the titration syringe, was sequentially injected into the reaction chamber containing the METTL14 protein. Each injection was performed at 120 s intervals with a stirring speed of 500 rpm. The thermodynamic response was monitored using high-feedback mode, and the raw calorimetric data were analyzed using the MicroCal PEAQ-ITC analysis software.

### Surface plasmon resonance (SPR) assay

Analysis of direct interactions between GDPPH1 and METTL14 was performed at 25 °C on a Biacore S200 SPR instrument (GE Healthcare). SPR running buffer contained PBS (pH 7.4) supplemented with 5% DMSO and was prepared immediately before measurement. Recombinant human protein METTL14 was immobilized via amine coupling on a flow cell of the chip. The remaining binding sites on the chips were blocked by 1 M ethanolamine (pH 8.5) at a flow rate of 10 uL/min for 7 min. Responses from a reference flow cell were subtracted to correct for nonspecific binding and bulk refractive index effects. GDPPH1, diluted in running buffer, was injected at different concentrations over both the reference and protein-immobilized flow cells at a flow rate of 30 uL/min for 180 s, followed by a dissociation phase of 300 s. The bound analytes were removed by a 30-s wash with running buffer.

### Immunohistochemistry (IHC)

Immunohistochemical staining was performed on paraffin-embedded tissue sections (5 µm thick). Sections were dried at 60 °C for 1 h, deparaffinized with xylene, and hydrated with graded ethanol solution. The sections were incubated with primary antibodies followed by HRP-conjugated secondary antibodies. DAB reagents were used as the substrate, and tissues were counterstained with hematoxylin at room temperature for 1 min. A negative control was included by omitting the primary antibodies. Images were captured using a digital microscope camera (E100) and analyzed with ImageJ software (version 2.0; National Institutes of Health).

### In vitro BBB model and permeability assays

The bEnd.3 cells (5 × 10^4^ cells/well) were seeded onto 24-well Transwell plates (Corning Life Sciences, Tewkesbury, MA, USA) with a pore size of 0.4 μm. The medium was refreshed every 1–2 days, and the cells were cultured for 5–7 days. Tight confluency was typically observed around day 7. Sodium fluorescein (NaF) transport was measured to evaluate whether the BBB model was successfully established. U251 cells were seeded in another 24-well plate at a density of 5 × 10^4^ cells/well and cultured for 24 h. The bEnd.3 monolayer in the upper chamber was transferred into the 24-well plate containing U251 cells. Subsequently, 500 μL of complete DMEM containing GDPPH1 at a concentration of 12 μM was added to the upper chamber and incubated for 48 h. Cell viability was evaluated using an MTT assay as described before.

### Statistical analysis

All experiments were performed at least three times, and the data were expressed as mean ± SD. Statistical analyses were conducted using GraphPad Prism 8.4.0 software. Kaplan–Meier survival analysis with a two-sided log-rank test was used to compare the overall survival between groups. Group differences were analyzed using the *t*-test and one-way ANOVA, followed by Tukey’s post hoc test for multiple comparisons. Statistical significance was defined as *P* < 0.05. Significant differences were indicated as *P < 0.05, **P < 0.01, or ***P < 0.001.

## Results

### GDPPH1 suppresses GBM cell proliferation and induces apoptosis

**Fig. 1:**
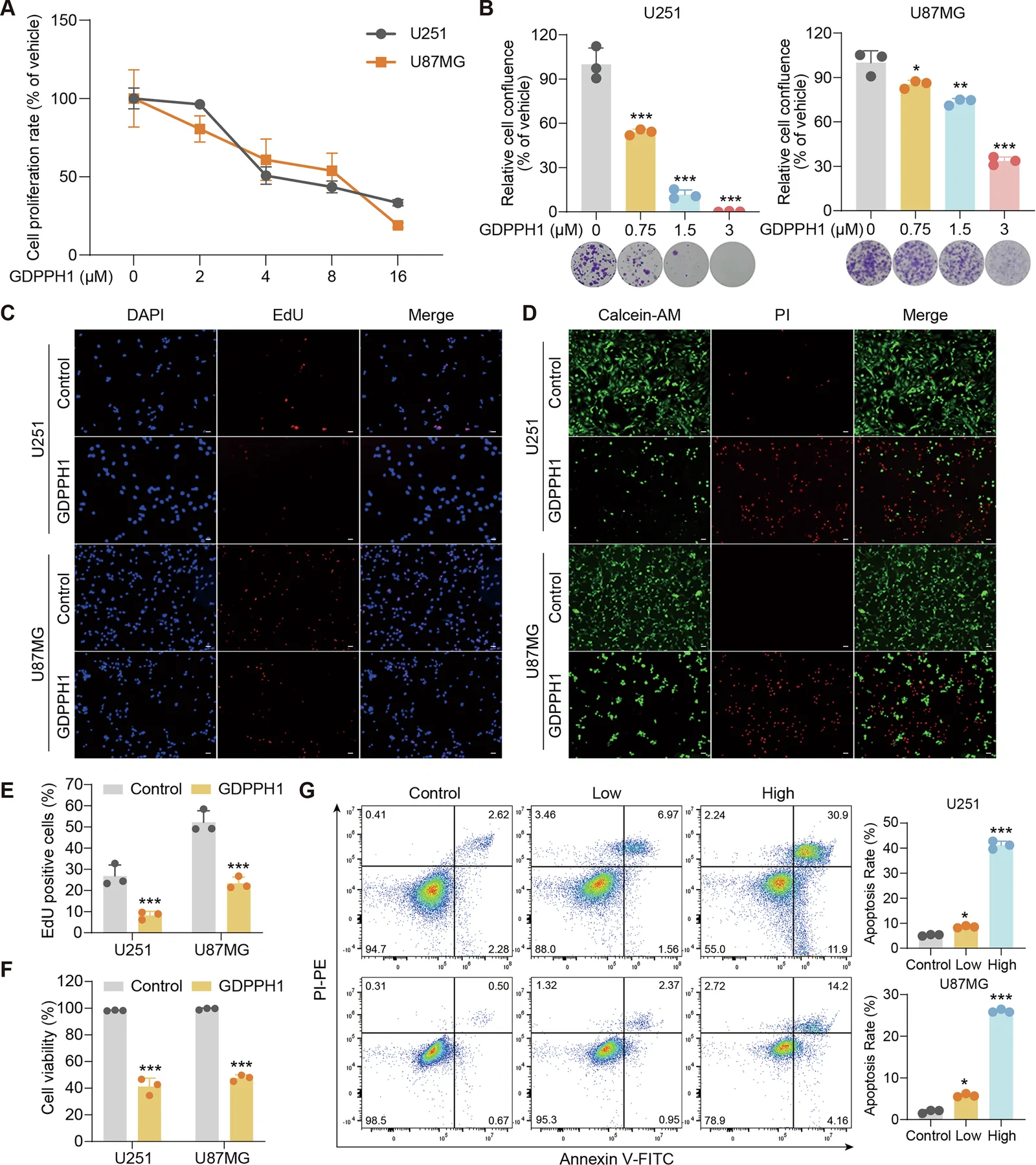
GDPPH1 suppresses GBM cell proliferation and induces apoptosis. **A** Quantification of inhibitory activity of GDPPH1 on cell viability in U251 and U87MG cell lines. The cell viability was determined by MTT assay, after treatment with various concentrations of GDPPH1 (2, 4, 8, 16 μM) for 48 h. **B** Plate colony formation of U251 and U87MG cells after GDPPH1 treatment for 14 days and quantitation of colony numbers. **C** Effects of GDPPH1 (1 μM) on DNA synthesis in U251 and U87MG cells at 24 h measured by ethynyl deoxyuridine (EdU). Scale bar, 50 μm. **D** Live and dead cell staining in U251 and U87MG cells. Scale bar, 50 μm. **E** Quantitative analysis of EdU in U251 and U87MG cells. **F** Quantitative analysis of live and dead cell staining in U251 and U87MG cells. **G** Flow cytometry analysis of cell death for U251 and U87MG cells treated with GDPPH1 for 24 h and subsequently stained with Annexin V and propidium iodide (PI). The results of the representative experiments and quantitative analysis are shown. Data are presented as mean ± SD from at least three independent replicates. Statistical significance between two groups was determined using an unpaired two-tailed Student’s *t*-test, while comparisons among multiple groups were performed using one-way ANOVA followed by Tukey’s post hoc test. *P* < 0.05 was considered statistically significant. *P < 0.05, **P < 0.01, ***P < 0.001.

Mangosteen has long been recognized for its therapeutic potential in treating various diseases, with its natural compounds garnering significant attention for their anti-tumor properties. In this study, nine candidate compounds isolated from mangosteen were screened, including symphonone I, WA76, garcinol (benzophenone), WA142-1-3, GDPPH1 (Garcim1), GDPPH2 (Garcim2), garmultin D, isoxanthochymol, and garcinialone (Supplementary Table 1). Among these, according to data from five GBM cell lines, GDPPH1 was identified as the most effective compound in suppressing GBM cell proliferation (Supplementary Fig. 2, Supplementary Table 5). Furthermore, the MTT assay demonstrated a concentration-dependent reduction in cell viability in U251 and U87MG cells treated with GDPPH1, with IC_50_ values of 6.73 μM and 6.37 μM, respectively (Fig. 1A). Colony formation assays also showed concentration-dependent inhibition of cell proliferation in U251 and U87MG cells (Fig. 1B). Similarly, EdU assay revealed a decrease of EdU-positive cells following GDPPH1 treatment (Fig. 1C, E). Moreover, calcein and propidium iodide (PI) staining showed reduced cell viability in GBM cells, as evidenced by an increase in red punctuate staining in the PI group (Fig. 1D, F). Flow cytometric analysis confirmed that GDPPH1 induced concentration-dependent increases in both early and late phase apoptotic cells (Fig. 1G). In contrast, no significant effects were observed on the migration, invasion, or stemness of GBM cells following GDPPH1 treatment (Supplementary Fig. 3). Collectively, these results demonstrate that GDPPH1 effectively suppresses GBM cell proliferation and induces apoptosis.

### GDPPH1 arrests the cell cycle at the G1/S phase by downregulation of CDK4 and Cyclin D1 in GBM cells

**Fig. 2:**
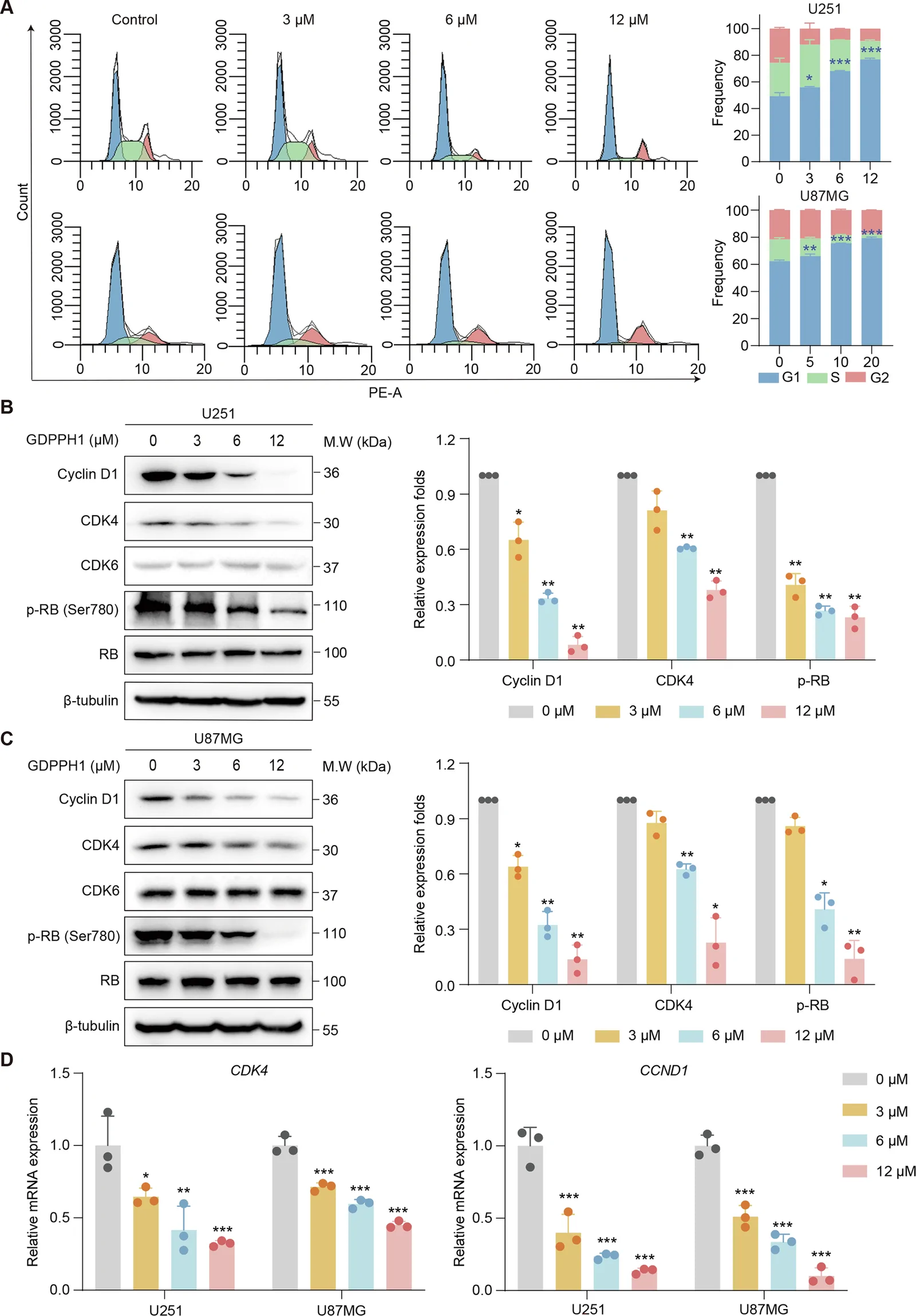
GDPPH1 arrests the cell cycle at the G1/S phase by downregulation of CDK4 and Cyclin D1 in GBM cells. **A** Effect of GDPPH1 on the cell cycle of U251 and U87MG cell lines assessed by PI staining-coupled flow cytometry. Quantitative analysis of concentration-dependent cell cycle arrest induced by GDPPH1 in U251 and U87MG cells. **B**, **C** GDPPH1 (3, 6, and 12 μM) treatment for 12 h decreased the protein expression of Cyclin D1, CDK4, CDK6, and p-RB (Ser780) in U251 and U87MG cell lines. β-tubulin was used as the loading control. **D** Relative mRNA expression of *CDK4* and *CCND1* in U251 and U87MG cell lines with 12 h GDPPH1 treatment at 3, 6, and 12 μM. Data are presented as mean ± SD from at least three independent replicates. Statistical significance among multiple groups was performed using one-way ANOVA followed by Tukey’s post hoc test. *P* < 0.05 was considered statistically significant. *P < 0.05, **P < 0.01, ***P < 0.001.

To explore the mechanism through which GDPPH1 inhibits the proliferation of GBM cells, we next investigated its effect on cell cycle progression, a critical determinant of cancer cell apoptosis and proliferation. Flow cytometry following propidium iodide (PI) staining revealed a significant concentration-dependent increase in the proportion of cells arrested at the G1/S phase upon GDPPH1 treatment (Fig. 2A). Since the CDK4/Cyclin D1-RB pathway, a key regulator of the G1-to-S phase transition, are frequently observed in GBM [23], we then examined the effect of GDPPH1 on CDK4/Cyclin D1/RB axis in GBM cells. Notably, CDK4 and Cyclin D1, as well as the phosphorylated RB protein levels, were downregulated in U251 (Fig. 2B), U87MG (Fig. 2C), LN229, and U118MG (Supplementary Fig. 4A, B), while the levels of other G1/S phase regulators, including p21, p27, CDK2, Cyclin E1, and E2F1 remained unchanged (Supplementary Fig. 4C). Further analysis revealed that GDPPH1 downregulated *CDK4* and *CCND1* mRNA expression in a concentration-dependent manner (Fig. 2D, Supplementary Fig. 4D), while their protein stability was unaffected (Supplementary Fig. 5A, B). These data demonstrate that GDPPH1 induces cell cycle arrest in GBM cells through downregulation of CDK4 and Cyclin D1 at the mRNA level.

**Fig. 3:**
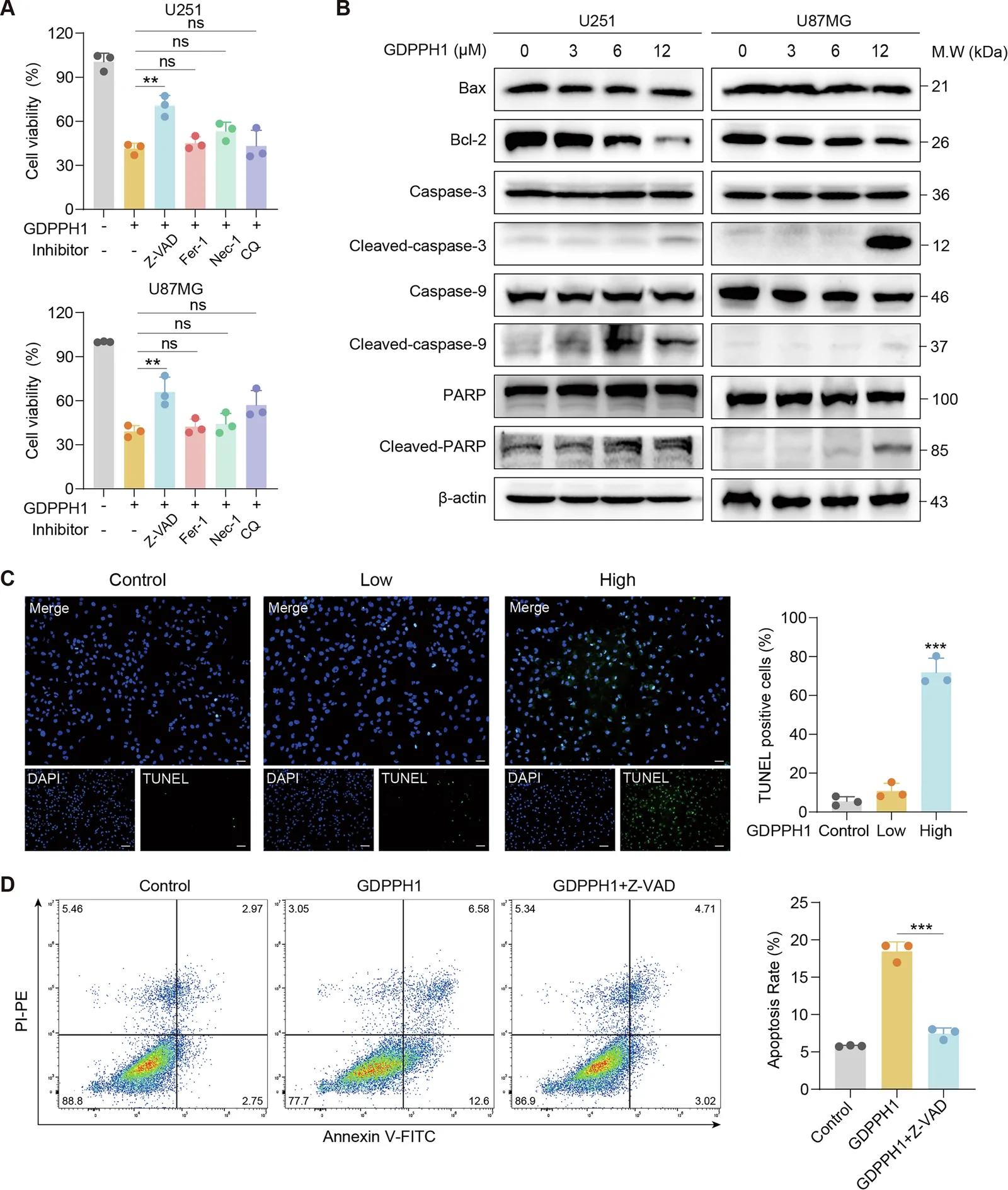
GDPPH1 induces cell apoptosis in GBM cells. **A** U251 and U87MG cells treated with different cell death inhibitors, including Z-VAD-FMK (pan-caspase inhibitor, Z-VAD), Ferrrostatin-1 (ferroptosis inhibitor, Fer-1), Necrostatin-1 (necrosis inhibitor, Nec-1), and Chloroquine (autophagy inhibitor, CQ), in the presence of GDPPH1 (8 μM) treatment. **B** Western blot to detect the expression of apoptosis-related proteins in GBM cells after 18 h treatment with GDPPH1 (3, 6, 12 μM). **C** TUNEL staining reveals increased apoptosis in GDPPH1-treated tumors. Scale bars, 50 μm in merged images and 100 μm in individual channel images. **D** Flow cytometry analysis of cell apoptosis for U87MG cells treated with GDPPH1 (8 μM) with or without Z-VAD (10 μM) for 48 h and subsequently stained with Annexin V and PI. Data are presented as mean ± SD from at least three independent replicates. Statistical significance among multiple groups was performed using one-way ANOVA followed by Tukey’s post hoc test. *P* < 0.05 was considered statistically significant. *P < 0.05, **P < 0.01, ***P < 0.001.

To determine the primary mode of cell death induced by GDPPPH1 in GBM cells, we employed specific inhibitors of apoptosis (Z-VAD-FMK, Z-VAD), ferroptosis (Ferrostatin-1, Fer-1), necrosis (Necrostatin-1, Nec-1), and autophagy (Chloroquine, CQ). Among these, only Z-VAD significantly rescued GDPPH1-induced cell death (Fig. 3A), suggesting that apoptosis is the predominant form of cell death induced by GDPPH1. Consistent with this, Western blot analysis revealed a concentration-dependent decrease in the anti-apoptotic protein Bcl-2 and increased cleavage of caspase-3, caspase-9, and PARP into their active forms (Fig. 3B, Supplementary Fig. 6). TUNEL staining further showed a marked increase in DNA fragmentation in GDPPH1-treated cells (Fig. 3C). Furthermore, Annexin/PI flow cytometry confirmed that Z-VAD treatment significantly reduced apoptosis in GDPPH1-treated GBM cells (Fig. 3D).

Collectively, these results demonstrate that GDPPH1 induces cell cycle arrest through downregulation of CDK4 and Cyclin D1, which subsequently leads to apoptosis in GBM cells.

### GDPPH1 suppresses *CDK4* and *CCND1* mRNA levels in an m^6^A-dependent manner

**Fig. 4:**
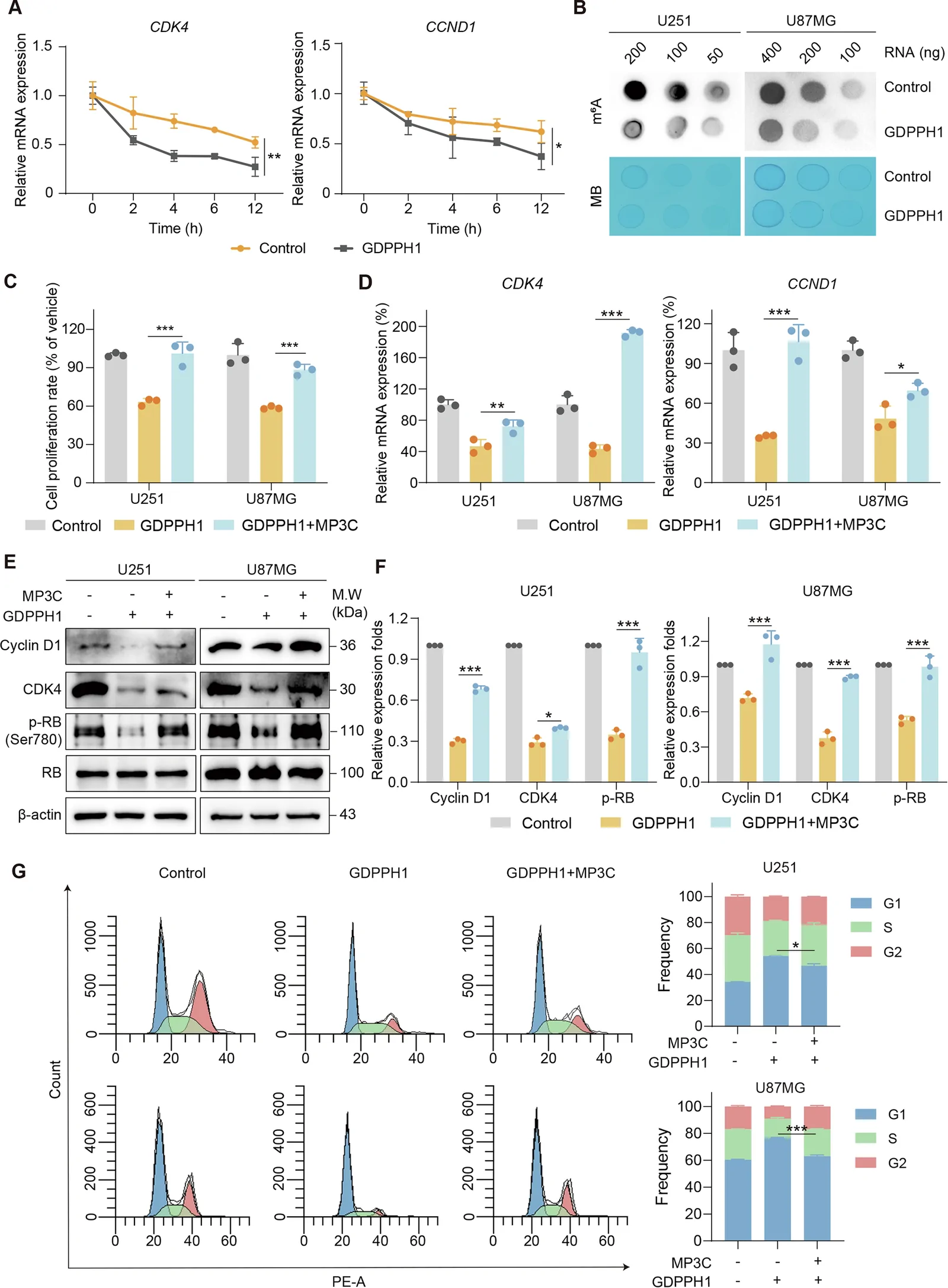
GDPPH1 suppresses CDK4 and *CCND1* mRNA levels in an m^6^A-dependent manner. **A** Stability of *CDK4* and *CCND1* mRNA upon GDPPH1 treatment on U251 cells examined by actinomycin D (Act D) chase assay. **B** The m^6^A level of total RNA isolated from GDPPH1-treated U251 and U87MG cells was indicated by m^6^A dot blot. **C** In U251 and U87MG cells treated with GDPPH1 and/or MP3C, cell viability was measured by MTT. **D** qPCR analysis of *CDK4* and *CCND1* in U251 and U87MG cells upon GDPPH1 and/or MP3C. **E** Western blot analysis of Cyclin D1, CDK4, and p-RB (Ser780) in U251 and U87MG cells upon GDPPH1 and/or MP3C. **F** Quantification of the expression of indicated protein levels in (**E**). **G** Cell cycle analysis of U251 and U87MG cells upon GDPPH1 and/or MP3C. Data are presented as mean ± SD from at least three independent replicates. Statistical significance among multiple groups was performed using one-way ANOVA followed by Tukey’s post hoc test. mRNA stability was analyzed using one-phase exponential decay analysis. *P* < 0.05 was considered statistically significant. *P < 0.05, **P < 0.01, ***P < 0.001.

To elucidate the regulatory mechanism through which GDPPH1 affects the mRNA levels of *CDK4* and *CCND1*, we first evaluated their mRNA stability. GDPPH1 treatment significantly reduced the stability of *CDK4* and *CCND1* mRNA in U251 cells (Fig. 4A). Given the established role of *N*^6^-methyladenosine (m^6^A) in regulating RNA stability, we hypothesized that GDPPH1 might exert its effects through m^6^A-dependent mechanisms. Dot blot assays confirmed that GDPPH1 treatment reduced the global m^6^A level in U251 and U87MG cells compared to controls (Fig. 4B). Using the SRAMP database, we identified high-confidence m^6^A modification sites in *CDK4* and *CCND1* mRNAs (Supplementary Fig. 7A). Based on this information, we designed targeted primers for MeRIP-qPCR assays, which revealed that the RNA m^6^A levels of specific positions within *CDK4* and *CCND1* were significantly enriched (Supplementary Fig. 7B).

To further validate the involvement of m^6^A modifications, we utilized methyl piperidine-3-carboxylate (MP3C), an agonist of the METTL3-METTL14-WTAP complex [24, 25]. Co-treatment with MP3C could effectively rescue the inhibition of proliferation induced by GDPPH1 in GBM cells (Fig. 4C). Co-treatment with MP3C also rescued the GDPPH1-induced reductions in mRNA levels of *CDK4* and *CCND1* (Fig. 4D), and their protein levels were similarly reversed (Fig. 4E, F). Additionally, MP3C treatment reversed the G1/S phase arrest induced by GDPPH1 in both U251 and U87MG cells (Fig. 4G). These findings indicate that GDPPH1 downregulates CDK4 and Cyclin D1 expression through m^6^A-dependent mechanisms.

### GDPPH1-regulated CDK4 and Cyclin D1 expression is associated with downregulation of METTL14

**Fig. 5:**
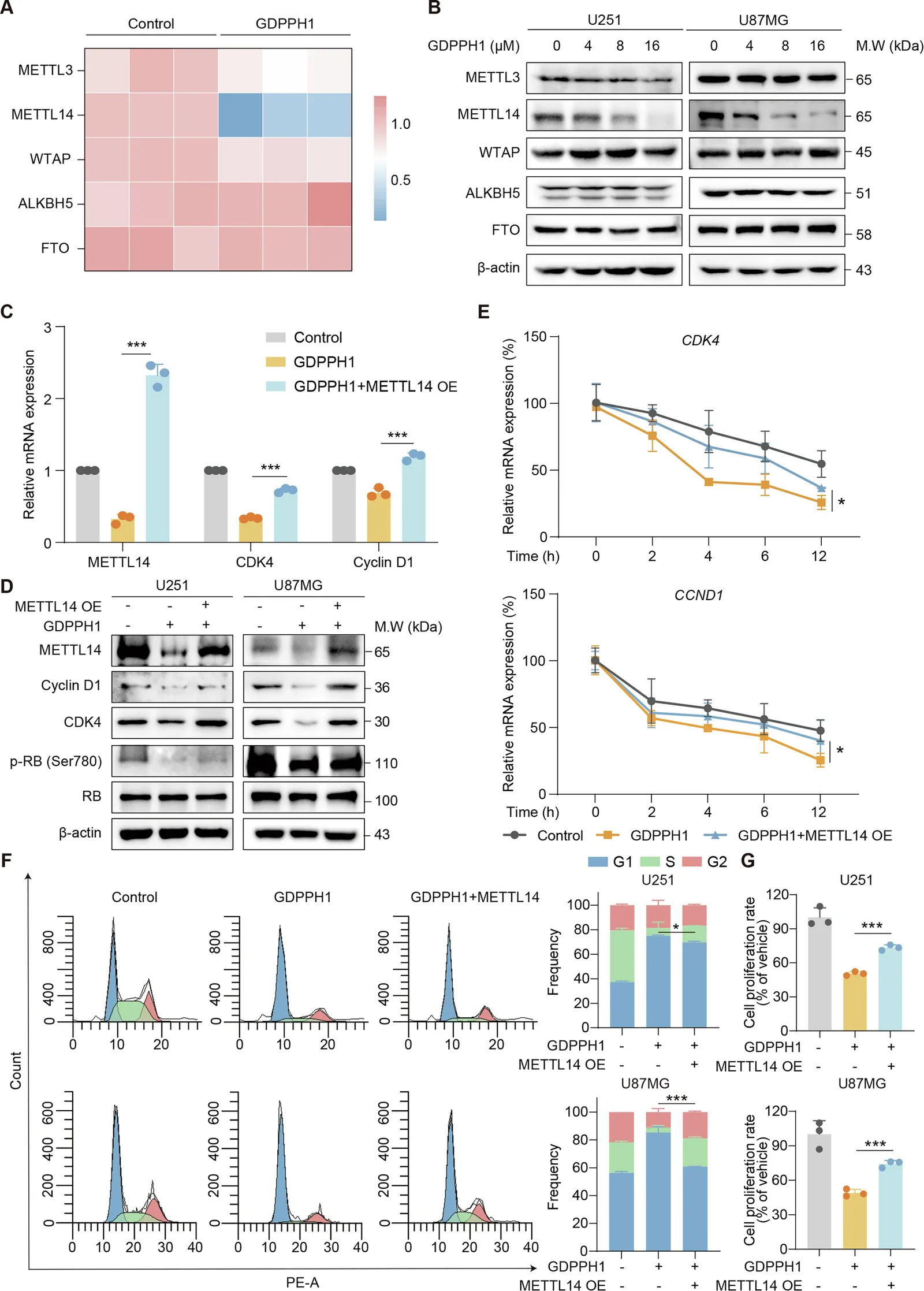
GDPPH1-regulated CDK4 and Cyclin D1 expression is primarily associated with METTL14. **A** qPCR analysis of key m^6^A-related enzyme in U251 and U87MG cells following GDPPH1 treatment. **B** Western blot analysis of METTL3, METTL14, WTAP, ALKBH5, and FTO in U251 and U87MG cells upon GDPPH1 treatment. **C** qPCR analysis of *METTL14*, *CDK4*, and *CCND1* in U251 and U87MG cells following GDPPH1 treatment with or without METTL14 overexpression (OE). **D** Western blot analysis of Cyclin D1, CDK4, and p-RB (Ser780) in U251 and U87MG cells under GDPPH1 treatment with or without METTL14 OE. **E** Stability of *CDK4* and *CCND1* mRNA upon GDPPH1 treatment with or without METTL14 OE, assessed by Act D chase assay. **F** Cell cycle distribution of U251 and U87MG cells following GDPPH1 treatment with or without METTL14 OE. **G** Cell viability was measured by MTT assay in U251 and U87MG cells treated with GDPPH1 in the presence or absence of METTL14 OE. Data are presented as mean ± SD from at least three independent replicates. Statistical significance between two groups was determined using an unpaired two-tailed Student’s *t*-test, while comparisons among multiple groups were performed using one-way ANOVA followed by Tukey’s post hoc test. mRNA stability was analyzed using one-phase exponential decay analysis. *P* < 0.05 was considered statistically significant. *P < 0.05, **P < 0.01, ***P < 0.001.

The degradation of mRNA regulated by m^6^A modification is largely influenced by m^6^A methyltransferases such as METTL3 and METTL14, and demethylases like ALKBH5 and FTO [26]. We hypothesized that GDPPH1-induced dysregulation of the m^6^A modification might stem from alterations in the expression of these key m^6^A enzymes. RT-qPCR and Western blot analyses revealed that the levels of METTL14 were significantly reduced following GDPPH1 treatment in GBM cells (Fig. 5A, B, Supplementary Fig. 8A, B). To investigate whether METTL14 plays a functional role in GDPPH1’s effects, RNA immunoprecipitation (RIP) assays were performed, and the results demonstrated that both *CDK4* and *CCND1* mRNAs were significantly enriched in METTL14 immunoprecipitates, indicating a direct association between METTL14 and these transcripts (Supplementary Fig. 8C, D). Overexpressed METTL14 in U251 and U87MG cells and subsequently treated the cells with GDPPH1. At the mRNA levels, overexpression of METTL14 rescued the GDPPH1-induced decrease of *CDK4* and *CCND1* (Fig. 5C). Similarly, METTL14 overexpression largely counteracted GDPPH1-mediated suppression of CDK4, Cyclin D1, and phospho-RB (Ser780) expression (Fig. 5D, Supplementary Fig. 8E, F). Importantly, METTL14 overexpression also increased the mRNA stability of *CDK4* and *CCND1* upon GDPPH1 treatment (Fig. 5E). Furthermore, GDPPH1-induced cell cycle arrest and proliferation were notably reversed in the METTL14-overexpression group (Fig. 5F, G). These findings indicate that METTL14 plays a crucial role in the suppression of GBM progression induced by GDPPH1.

### The m^6^A-dependent downregulation of CDK4 and Cyclin D1 by GDPPH1 is mediated by YTHDF2

**Fig. 6:**
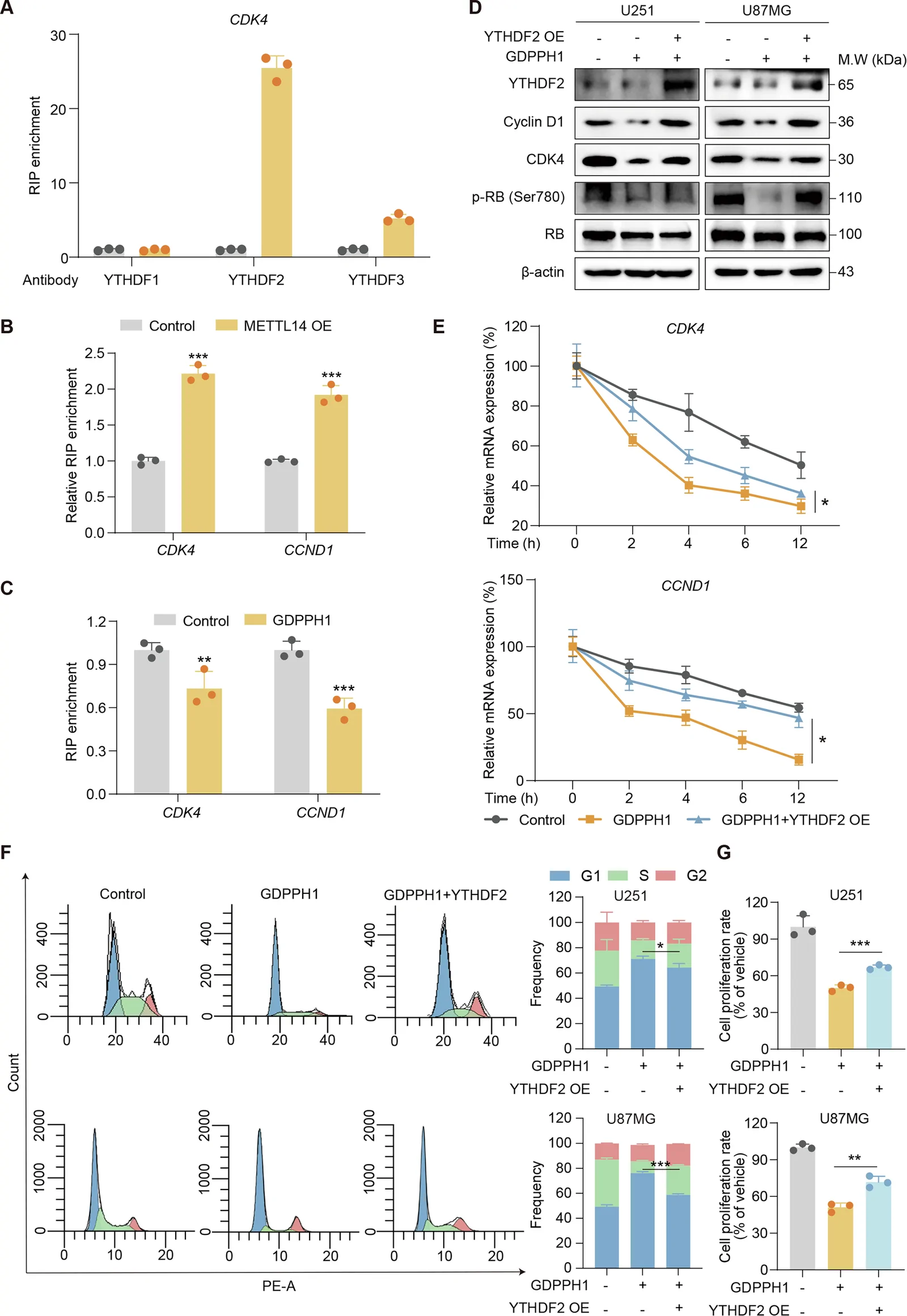
The m^6^A-dependent downregulation of CDK4 and Cyclin D1 by GDPPH1 is associated with YTHDF2. **A** Interaction between YTHDF1, YTHDF2, YTHDF3, and *CDK4* detected by RIP. **B** Interaction between YTHDF2 and *CDK4*, *CCND1*, with METTL14 OE detected by RIP. **C** The interaction between YTHDF2 and *CDK4*, *CCND1* were diminished by GDPPH1. **D** Western blot analysis of Cyclin D1, CDK4, and p-RB (Ser780) in U251 and U87MG cells upon GDPPH1 with or without YTHDF2 OE. **E** Stability of *CDK4* and *CCND1* mRNA upon GDPPH1 treatment with or without YTHDF2 OE examined by Act D chase assay. **F** Cell cycle analysis and cell viability of U251 and U87MG cells upon GDPPH1 with or without YTHDF2 OE. **G** U251 and U87MG cells treated with GDPPH1 and/or YTHDF2 OE, cell viability was measured by MTT. Data are presented as mean ± SD from at least three independent replicates. Statistical significance between two groups was determined using an unpaired two-tailed Student’s *t*-test, while comparisons among multiple groups were performed using one-way ANOVA followed by Tukey’s post hoc test. mRNA stability was analyzed using one-phase exponential decay analysis. *P* < 0.05 was considered statistically significant. *P < 0.05, **P < 0.01, ***P < 0.001.

m^6^A modifications, added by m^6^A methyltransferases, are recognized by m^6^A “reader” proteins, which influence gene expression by promoting or inhibiting mRNA degradation, such as YTHDF1, YTHDF2, and YTHDF3 [27, 28]. To identify which “reader” directly binds to *CDK4* and *CCND1* mRNA, we performed RNA immunoprecipitation (RIP) assays. The RIP results demonstrated that anti-YTHDF2 antibodies co-immunoprecipitated significantly more *CDK4* mRNA compared to anti-YTHDF1 or anti-YTHDF3 antibodies (Fig. 6A). *CCND1* mRNA was also significantly enriched in anti-YTHDF2 immunoprecipitation (Supplementary Fig. 9A). YTHDF2 has been reported to be recruited by METTL14 to mediate mRNA decay [29]. Indeed, the binding between YTHDF2 and *CDK4* or *CCND1* were enhanced when METTL14 was overexpressed (Fig. 6B). However, the binding between YTHDF2 and these mRNAs was diminished upon GDPPH1 treatment (Fig. 6C). Further investigation revealed that overexpression of YTHDF2 in GBM cells elevated the protein levels of CDK4, Cyclin D1, and phospho-RB (Fig. 6D, Supplementary Fig. 9B, C), while also increasing the mRNA stability of *CDK4* and *CCND1* (Fig. 6E). Flow cytometry and cell proliferation assay confirmed that overexpression YTHDF2 rescued GDPPH1-induced cell cycle arrest, reversing the inhibitory effects of GDPPH1 on GBM cell proliferation (Fig. 6F, G). These findings demonstrate that GDPPH1 modulates CDK4 and Cyclin D1 expression through METTL14-mediated, YTHDF2-dependent m^6^A modification.

### METTL14 is the direct target of GDPPH1

**Fig. 7:**
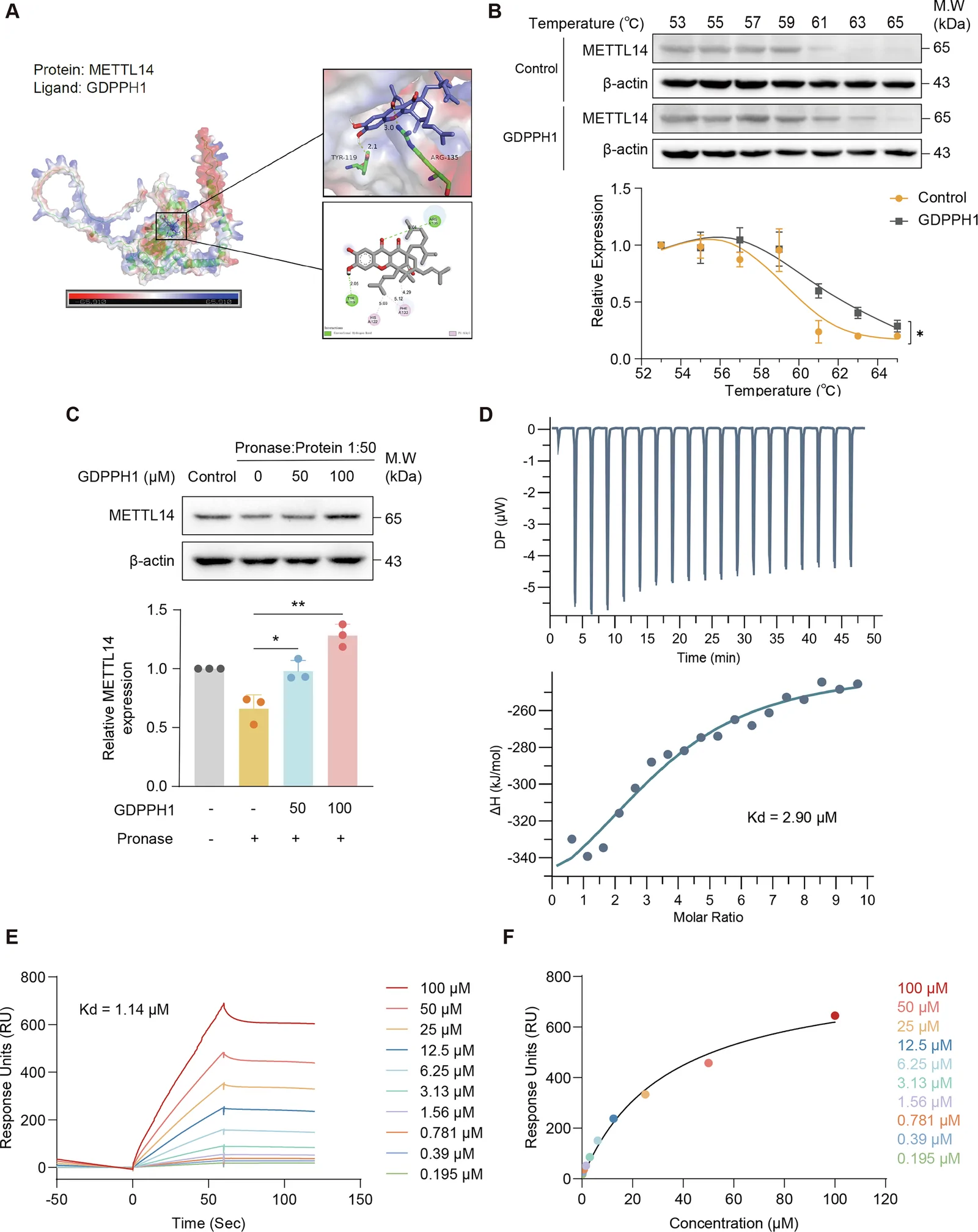
METTL14 is the direct target of GDPPH1. **A** Molecular docking analysis revealed that GDPPH1 forms a stable complex with METTL14, with a binding energy (ΔG) of −7.5 kcal/mol. **B**, **C** CETSA and DARTS assays confirm a direct and specific interaction between GDPPH1 and METTL14. **D** The binding affinity between GDPPH1 and METTL14 was determined using ITC. **E** The interaction between GDPPH1 and METTL14 protein was measured by SPR. **F** Steady-state affinity analysis based on equilibrium response units plotted against analyte concentration. Data are presented as mean ± SD from at least three independent replicates. Statistical significance among multiple groups was performed using one-way ANOVA followed by Tukey’s post hoc test. *P* < 0.05 was considered statistically significant. *P < 0.05, **P < 0.01, ***P < 0.001.

To fully clarify the mechanism of the anti-GBM effects of GDPPH1, we first examined whether GDPPH1 exerts broad effects on canonical proliferation-related signaling pathways, including ERK, Akt, AMPK, β-catenin, and Hippo signaling. The results showed that GDPPH1 treatment did not induce significant changes in the activation or expression of the core components of these pathways (Supplementary Fig. 10). Based on these observations, we next sought to identify a more specific molecular target underlying the effects of GDPPH1. To gain mechanistic insight, we performed molecular docking analysis focusing on METTL14. The results revealed a strong interaction between GDPPH1 and METTL14, with a binding energy of −7.5 kcal/mol (Fig. 7A). To further validate this interaction in a cellular context, a Cellular Thermal Shift Assay (CETSA) was conducted. GDPPH1 treatment markedly increased the thermal stability of METTL14, as evidenced by a higher proportion of METTL14 protein remaining at elevated temperatures compared with the control group (Fig. 7B). Consistently, a drug affinity responsive target stability (DARTS) assay showed that GDPPH1 protected METTL14 from protease-induced degradation in a concentration-dependent manner, further supporting a direct interaction between GDPPH1 and METTL14 (Fig. 7C).

To further characterize the binding, isothermal titration calorimetry (ITC) and surface plasmon resonance (SPR) analyses were performed. ITC revealed a dissociation constant (Kd) of 2.90 μM for the GDPPH1-METTL14 interaction (Fig. 7D). Consistently, SPR analysis further supported this interaction with a comparable micromolar-range affinity and a concentration-dependent binding response (Fig. 7E, F), indicating a direct interaction between GDPPH1 and METTL14. Collectively, these results identify METTL14 as a direct target of GDPPH1.

### GDPPH1 suppresses GBM growth in vivo

**Fig. 8:**
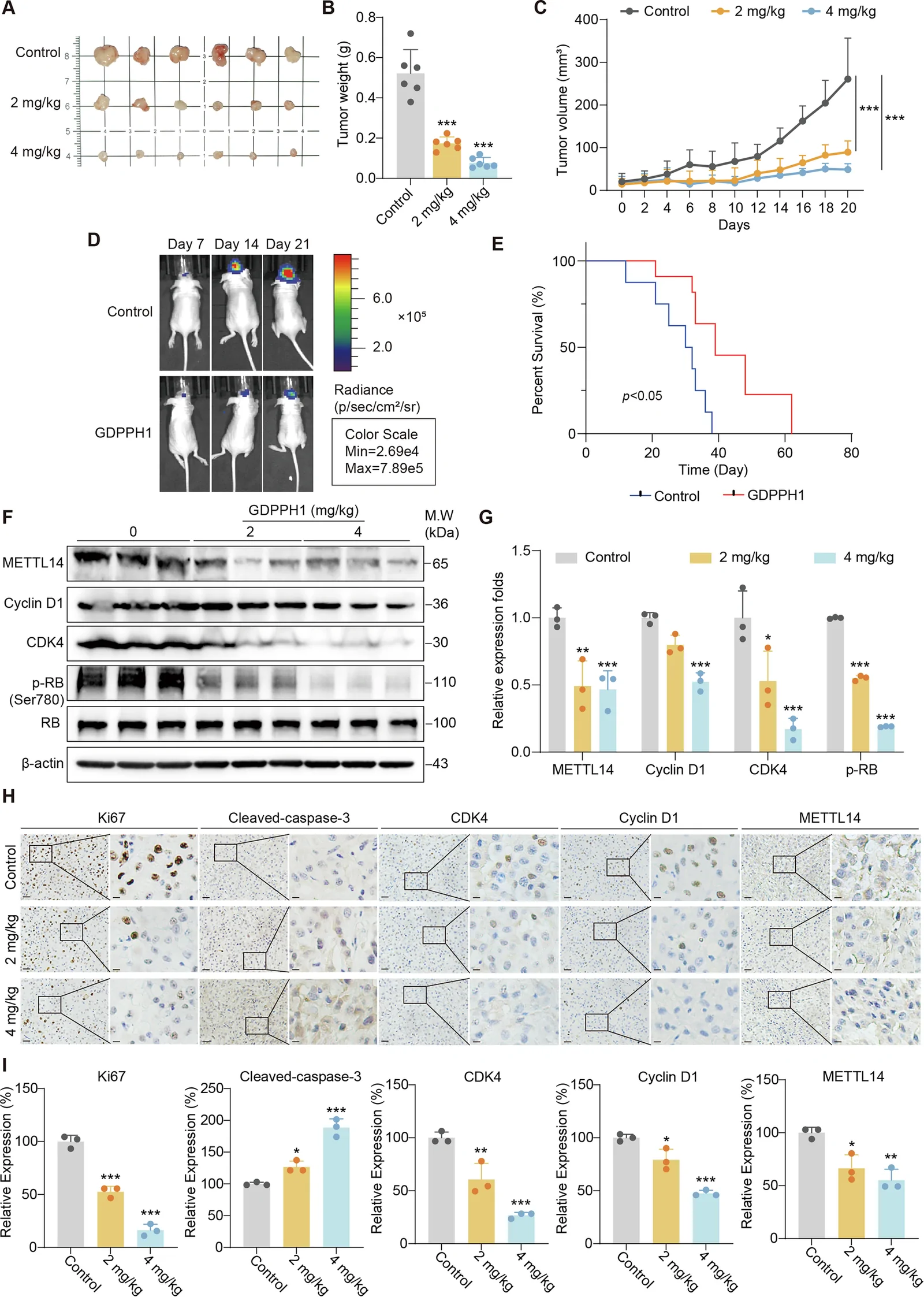
GDPPH1 suppresses GBM growth in vivo*.* **A** Images of tumors from the vehicle and GDPPH1 (2 and 4 mg/kg) treatment groups (*n* = 6). **B** GDPPH1 (2 and 4 mg/kg) treatment reduced the tumor weight. **C** Measurement of tumor volume in each group. **D** Representative images of bioluminescence of mice on days 7, 14, and 21 after implantation and quantitative analysis of these bioluminescence images for the vehicle and GDPPH1 treatment groups. **E** The overall survival (OS) of mice in the vehicle and GDPPH1-treated groups in the orthotopic model. **F** Measurement of protein expression levels of METTL14, Cyclin D1, CDK4, and p-RB (Ser780) in tumor tissues in GDPPH1 (2 and 4 mg/kg). **G** Quantification of the expression of indicated protein levels in (**F**). **H** Immunohistochemistry assay data showed that GDPPH1 (2 and 4 mg/kg) inhibited the protein expression of Ki67, METTL14, CDK4, Cyclin D1, and increased Cleaved-Caspase-3 in tumor tissues. Scale bars, 100 μm in lower-magnification views; 25 μm in higher-magnification views. **I** Quantification of IHC staining in (**H**). Data are presented as mean ± SD from at least three independent replicates. Statistical significance between two groups was determined using an unpaired two-tailed Student’s *t*-test, while comparisons among multiple groups were performed using one-way ANOVA followed by Tukey’s post hoc test. *P* < 0.05 was considered statistically significant. *P < 0.05, **P < 0.01, ***P < 0.001.

To evaluate the potential anti-GBM effect of GDPPH1 in vivo, we established both subcutaneous and intracranial tumor mouse models. Mice with subcutaneous xenograft tumors, generated using U251 cells, were randomly divided into three groups and administered GDPPH1 (2 mg/kg and 4 mg/kg) or vehicle via intraperitoneal injection every other day. Tumor size and weight measurements showed that GDPPH1 treatment significantly reduced tumor growth compared to the vehicle control group (Fig. 8A–C). In the intracranial xenograft model constructed with U87MG-luc cells, the GDPPH1-treated group exhibited slower tumor growth (Fig. 8D) and significantly longer survival (Fig. 8E) compared to the vehicle-treated group, further supporting the in vivo efficacy of GDPPH1 in GBM treatment.

Western blot analysis of tumor tissues showed that the expression of CDK4, Cyclin D1, phospho-RB (Ser780), and METTL14 was downregulated in the GDPPH1-treated group (Fig. 8F, G). Immunohistochemical staining of the tumor tissues revealed decreased expression of Ki67, CDK4, Cyclin D1, and METTL14, alongside an increase in cleaved-caspase 3 in GDPPH1-treated groups (Fig. 8H, I). In addition, we evaluated the potential systemic toxicity of GDPPH1 by monitoring body weight and performing blood biochemical, hematological, and histological analyses of major organs. Mice treated with GDPPH1 showed no significant loss in body weight compared with controls (Supplementary Fig. 11A). Furthermore, blood biochemical (Supplementary Fig. 11B–E) and hematological (Supplementary Fig. 11F–H), as well as histological examination of major organs (Supplementary Fig. 11I), revealed no significant pathological abnormalities. Together, these results suggest that GDPPH1 inhibits GBM tumors in vivo without significant toxicity.

## Discussion

Recent studies have highlighted the significant antitumor potential of natural compounds due to their relatively low systemic toxicity and their ability to modulate key oncogenic pathways. Recent studies have demonstrated that several phytochemicals [30, 31], including saponins, curcumin, and resveratrol, exert anti-glioma effects primarily by inducing cell cycle arrest and apoptosis [32–34]. However, the role of GDPPH1, a novel compound derived from mangosteen, in GBM remains unclear. In addition, most existing studies have focused on downstream signaling events, while the upstream regulatory mechanisms governing these effects remain incompletely understood. In this context, our study introduces an additional layer of regulation by demonstrating that GDPPH1 modulates GBM progression through the METTL14-m^6^A-YTHDF2 axis, which in turn influences the expression of key cell cycle regulators, CDK4 and Cyclin D1. These findings suggest that epitranscriptomic regulation may represent a previously underappreciated mechanism underlying the anti-tumor effects of natural compounds in glioblastoma.

Targeting cell cycle regulators has emerged as an effective strategy for cancer therapy. CDK4 and Cyclin D1 are critical regulators of the cell cycle. Analysis of TCGA and GTEx datasets showed that *CDK4* and *CCND1* are significantly upregulated in GBM tissues compared with normal brain tissues (Supplementary Fig. 12A, B). Aberrant CDK4 activation—often due to gene amplification or overexpression—has been implicated in various cancers, including GBM [35]. Furthermore, CDK4 expression showed a significant positive correlation with increasing WHO grade. CDK4/6 inhibitors, including palbociclib, ribociclib, and abemaciclib, have been investigated in GBM. However, their clinical translation remains challenging, and early studies of palbociclib in recurrent GBM have shown limited therapeutic benefit. In contrast, ribociclib and abemaciclib have demonstrated more promising preclinical activity, with ribociclib being evaluated in diffuse hemispheric glioma and in ongoing early-phase clinical trials in recurrent GBM [36].

Beyond the classical CDK4/Cyclin D1 cell cycle axis, our findings further implicate m^6^A RNA modification as an additional regulatory layer in GBM progression. Key m^6^A regulators, including METTL14 and YTHDF2, were associated with the expression pattern of the identified target genes, which were significantly enriched in recurrent GBM (Supplementary Fig. 12C–I). Elevated YTHDF2 expression has been linked to chemoresistance, malignant progression, and adverse clinical outcomes in GBM [37]. This suggests that epitranscriptomic regulation may contribute to the activation of cell cycle-related signaling in tumor recurrence. Therefore, our results expand the current understanding of CDK4/Cyclin D1-associated tumor biology by incorporating an m^6^A-mediated regulatory dimension and may provide a theoretical basis for exploring epitranscriptome-targeted strategies in combination with cell cycle inhibition in GBM.

METTL14, a core component of the m^6^A “writer” complex, and YTHDF2, a major m^6^A reader protein, are critical regulators of post-transcriptional gene expression and have been implicated in cancer progression through the modulation of mRNA stability. Previous studies have shown that METTL14 can regulate the expression of cell cycle-related genes, including CDK4, via m^6^A-dependent mechanisms. For example, in prostate cancer, METTL14 overexpression has been reported to increase m^6^A modification on *CDK4* mRNA, leading to accelerated transcript degradation and reduced *CDK4* expression, thereby inducing G1 phase arrest [38]. In parallel, YTHDF2, although classically known to mediate mRNA decay, has been reported to exhibit context-dependent regulatory functions in tumor cells, including the modulation of cell cycle-related genes expression [18, 21, 39]. Despite these findings, the roles of METTL14 and YTHDF2 in regulating CDK4 and Cyclin D1 expression in GBM remain poorly defined. In the present study, we demonstrated that overexpression of either METTL14 or YTHDF2 partially reversed GDPPH1-induced G1/S phase arrest, indicating that the m^6^A regulatory machinery is functionally involved in mediating the anti-proliferative effects of GDPPH1.

Building upon these observations, we further sought to determine whether GDPPH1 directly targets components of the m^6^A regulatory machinery. Molecular docking analysis predicted a favorable interaction between GDPPH1 and METTL14. This interaction was subsequently supported by multiple orthogonal approaches, including CETSA and DARTS assays, which demonstrated enhanced thermal stability and protease resistance of METTL14 upon GDPPH1 treatment. Furthermore, biophysical analyses using ITC and SPR confirmed a direct interaction between GDPPH1 and METTL14 with micromolar-range binding affinity. Taken together with the rescue experiments described above, these findings indicate that METTL14 is not only functionally involved but also serves as a direct molecular target of GDPPH1 in GBM cells.

Interestingly, in addition to its direct interaction with METTL14, GDPPH1 treatment also resulted in altered METTL14 expression levels. While small molecule-protein interactions are typically associated with modulation of protein activity, the observed changes in METTL14 abundance may reflect additional regulatory mechanisms at the transcriptional or post-translational level [40, 41]. Further investigation is required to determine whether this effect is mediated directly or indirectly.

The clinical application of many candidate drugs for GBM is hindered by their insufficient penetration across the blood–brain barrier (BBB). In this study, both theoretical considerations and experimental evidence were used to evaluate the potential BBB permeability of GDPPH1. From a physicochemical perspective, GDPPH1 possesses a relatively low molecular weight, a limited number of hydrogen bond donors, and high lipophilicity, features generally favorable for passive diffusion across the lipid-rich BBB. Experimentally, the anti-GBM efficacy observed in the bEnd.3 in vitro BBB model (Supplementary Fig. 13) and the orthotopic GBM animal model (Fig. 8D) provides additional evidence supporting the potential BBB permeability of GDPPH1.

**Fig. 9:**
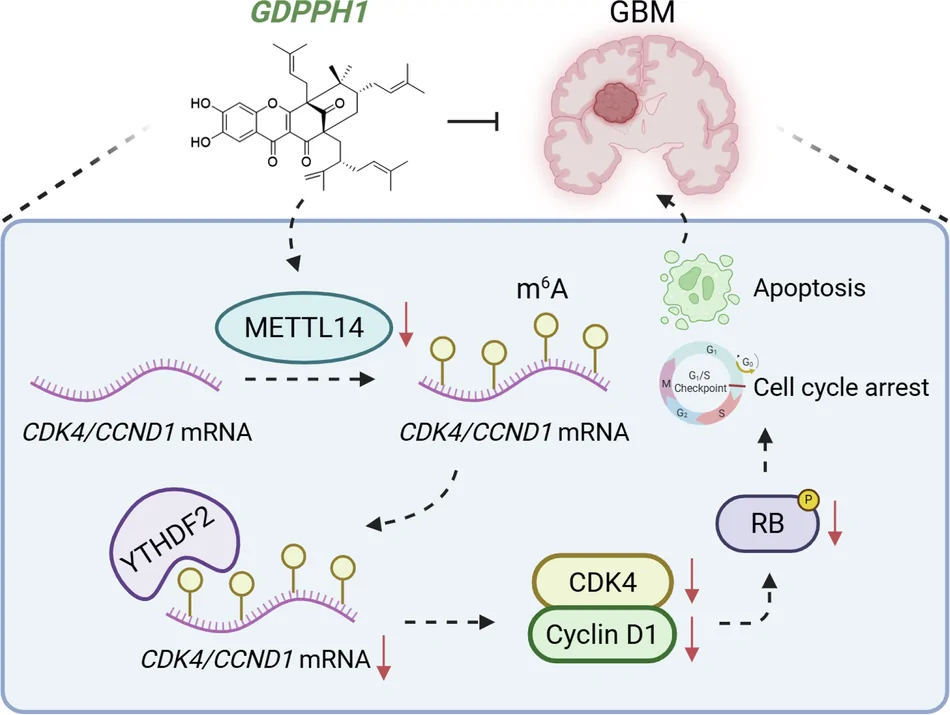
Schematic illustration of GDPPH1 induces cell cycle arrest at G1/S phase through regulation of YTHDF2-dependent *CDK4/CCND1* mRNA degradation in glioblastoma.

In summary, our study reveals a novel mechanism by which the natural product GDPPH1 targets m^6^A methylation regulators to suppress GBM proliferation. Specifically, GDPPH1 downregulates CDK4 and Cyclin D1 expression via the METTL14-YTHDF2 axis, leading to cell cycle arrest and subsequent apoptosis (Fig. 9). These findings offer new therapeutic insights into targeting the m^6^A epitranscriptome in GBM and highlight the potential of GDPPH1 as a promising candidate for GBM treatment.

## Supplementary information

Supplementary Figure 1

Supplementary Figure 2

Supplementary Figure 3

Supplementary Figure 4

Supplementary Figure 5

Supplementary Figure 6

Supplementary Figure 7

Supplementary Figure 8

Supplementary Figure 9

Supplementary Figure 10

Supplementary Figure 11

Supplementary Figure 12

Supplementary Figure 13

Supplementary Figure legends

Supplementary Table 1 to Supplementary Table 5

Checklist

Uncropped Western Blots
