# Supplementary material for: GDPPH1 induces cell cycle arrest by downregulation of CDK4/Cyclin D1 via the m6A-METTL14-YTHDF2 axis, attenuating glioblastoma progression
Source: Cell Death Dis. 2026 Jul 3;17(1):828. doi: 10.1038/s41419-026-09036-x (PMC13601516; doi:10.1038/s41419-026-09036-x)
Supplement: Supplementary file 1 [file 41419_2026_9036_MOESM10_ESM.pdf]

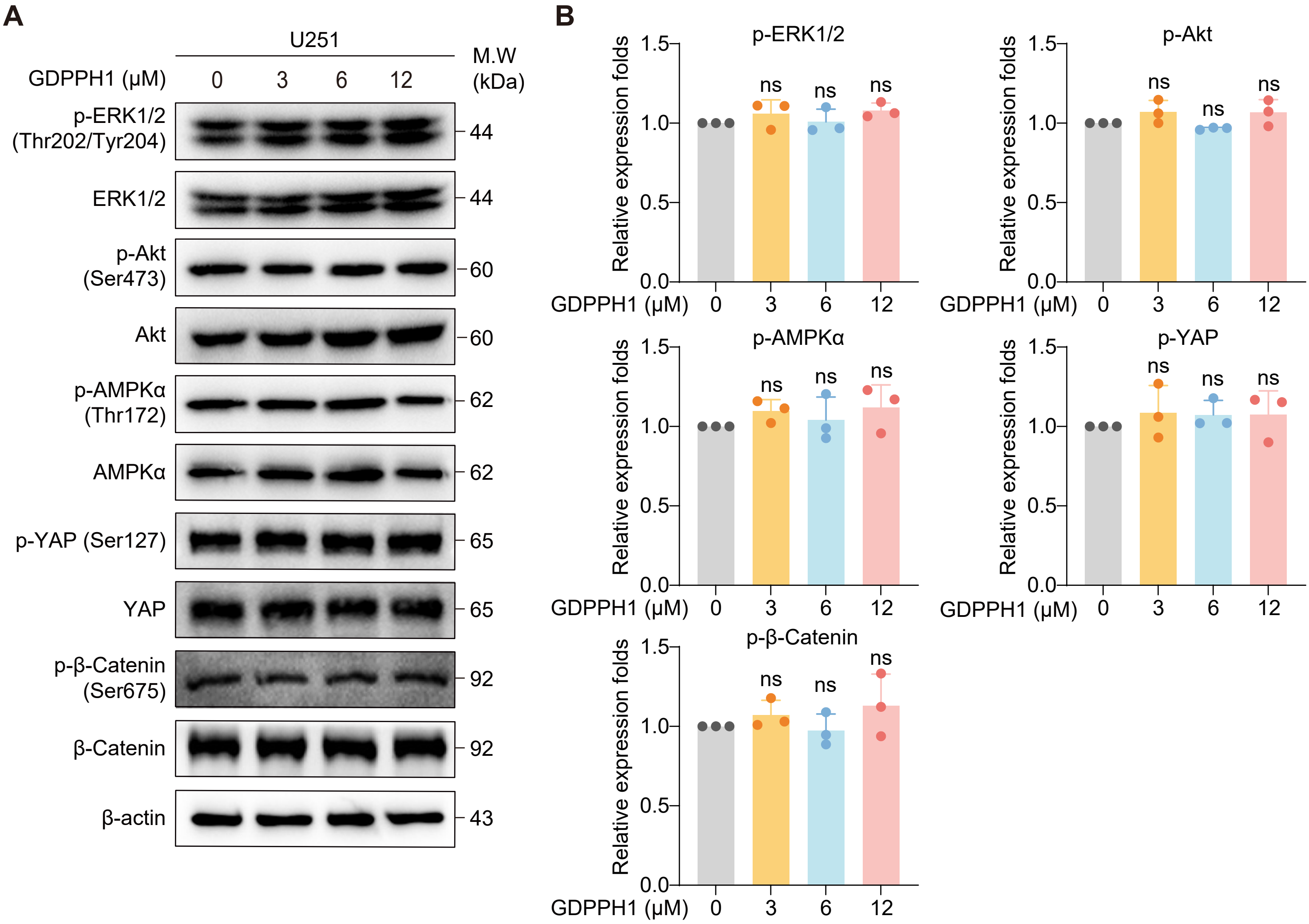
A
U251
GDPPH1 (μM) 0 3 6 12
M.W (kDa)
p-ERK1/2 (Thr202/Tyr204) 44
ERK1/2 44
p-Akt (Ser473) 60
Akt 60
p-AMPKα (Thr172) 62
AMPKα 62
p-YAP (Ser127) 65
YAP 65
p-β-Catenin (Ser675) 92
β-Catenin 92
β-actin 43
B
p-ERK1/2
p-Akt
p-AMPKα
p-YAP
p-β-Catenin
Relative expression folds
1.5
1.0
0.5
0.0
ns
GDPPH1 (μM) 0 3 6 12
