# Supplementary material for: GDPPH1 induces cell cycle arrest by downregulation of CDK4/Cyclin D1 via the m6A-METTL14-YTHDF2 axis, attenuating glioblastoma progression
Source: Cell Death Dis. 2026 Jul 3;17(1):828. doi: 10.1038/s41419-026-09036-x (PMC13601516; doi:10.1038/s41419-026-09036-x)
Supplement: Supplementary file 2 [file 41419_2026_9036_MOESM11_ESM.pdf]

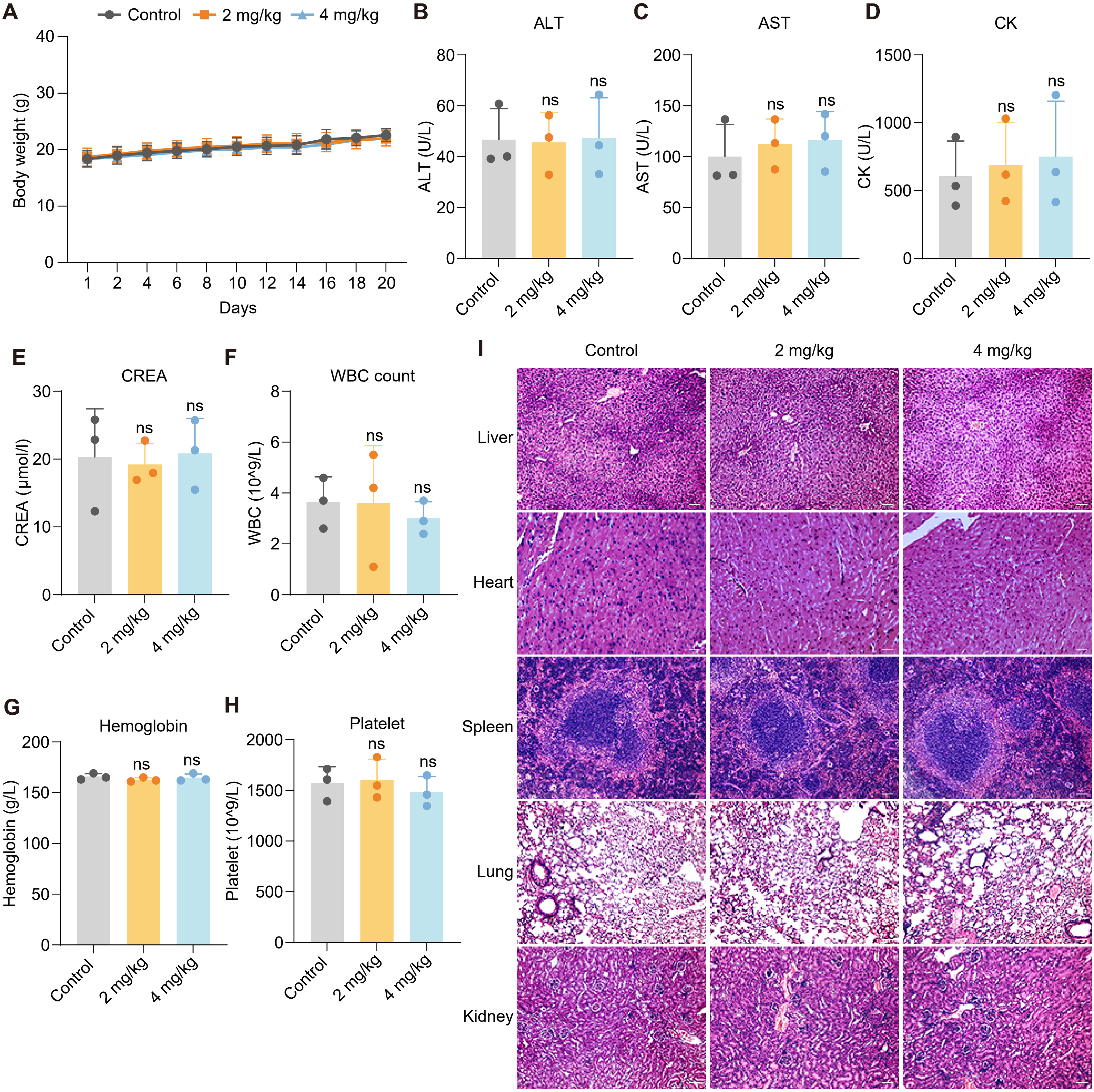

A
Control
2 mg/kg
4 mg/kg
Body weight (g)
Days
B
ALT
ALT (U/L)
C
AST
AST (U/L)
D
CK
CK (U/L)
E
CREA
CREA (μmol/l)
F
WBC count
WBC (10^9/L)
G
Hemoglobin
Hemoglobin (g/L)
H
Platelet
Platelet (10^9/L)
ns
I
Control
2 mg/kg
4 mg/kg
Liver
Heart
Spleen
Lung
Kidney
