# Supplementary material for: GDPPH1 induces cell cycle arrest by downregulation of CDK4/Cyclin D1 via the m6A-METTL14-YTHDF2 axis, attenuating glioblastoma progression
Source: Cell Death Dis. 2026 Jul 3;17(1):828. doi: 10.1038/s41419-026-09036-x (PMC13601516; doi:10.1038/s41419-026-09036-x)
Supplement: Supplementary file 3 [file 41419_2026_9036_MOESM12_ESM.pdf]

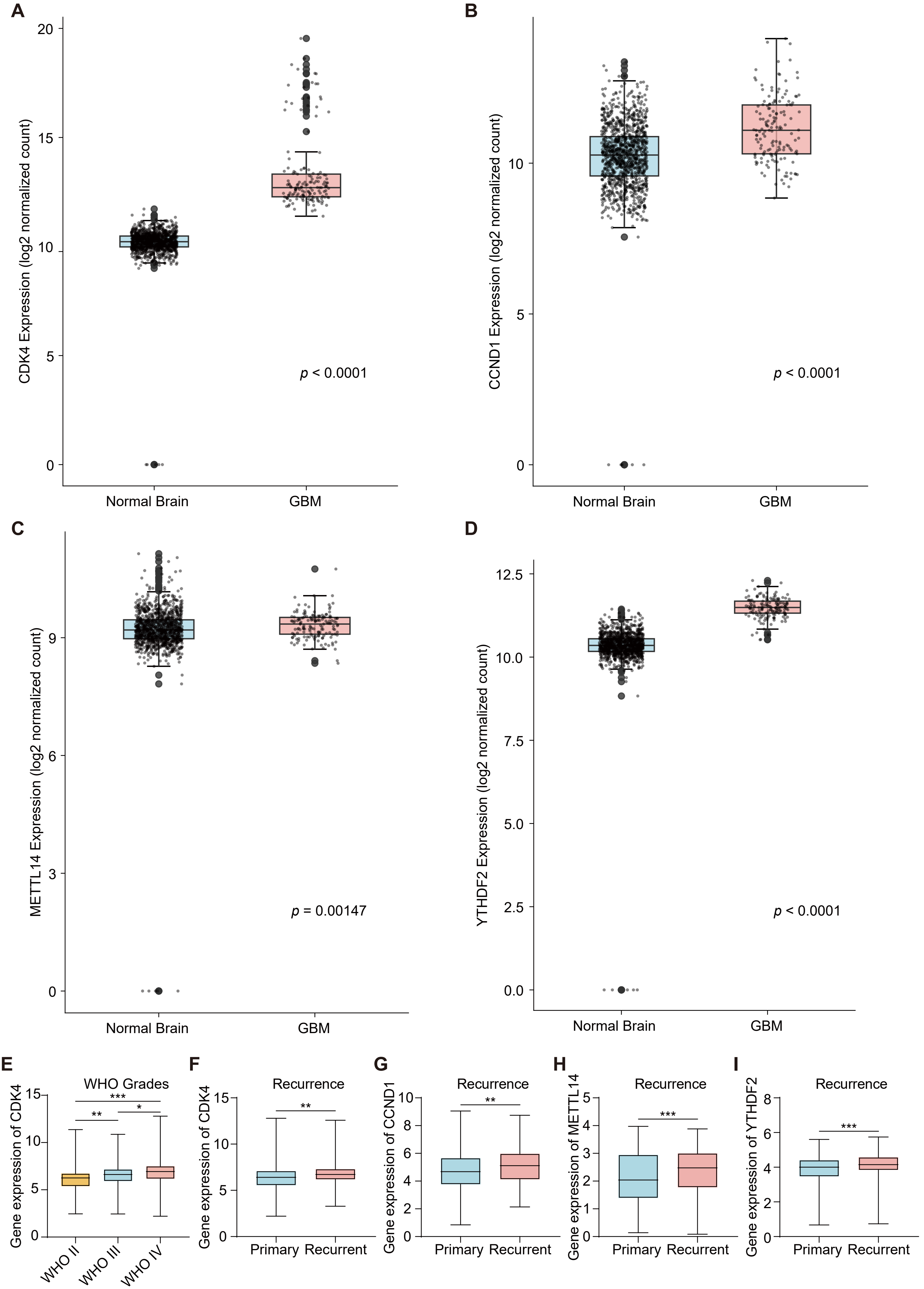

A
CDK4 Expression (log2 normalized count)
20
15
10
5
0
p < 0.0001
Normal Brain
GBM
B
CCND1 Expression (log2 normalized count)
10
5
0
p < 0.0001
Normal Brain
GBM
C
METTL14 Expression (log2 normalized count)
9
6
3
0
p = 0.00147
Normal Brain
GBM
D
YTHDF2 Expression (log2 normalized count)
12.5
10.0
7.5
5.0
2.5
0.0
p < 0.0001
Normal Brain
GBM
E
WHO Grades
Gene expression of CDK4
***
**
*
WHO II
WHO III
WHO IV
F
Recurrence
Gene expression of CDK4
**
Primary
Recurrent
G
Recurrence
Gene expression of CCND1
**
Primary
Recurrent
H
Recurrence
Gene expression of METTL14
***
Primary
Recurrent
I
Recurrence
Gene expression of YTHDF2
***
Primary
Recurrent
