# Supplementary material for: GDPPH1 induces cell cycle arrest by downregulation of CDK4/Cyclin D1 via the m6A-METTL14-YTHDF2 axis, attenuating glioblastoma progression
Source: Cell Death Dis. 2026 Jul 3;17(1):828. doi: 10.1038/s41419-026-09036-x (PMC13601516; doi:10.1038/s41419-026-09036-x)
Supplement: Supplementary file 4 [file 41419_2026_9036_MOESM13_ESM.pdf]

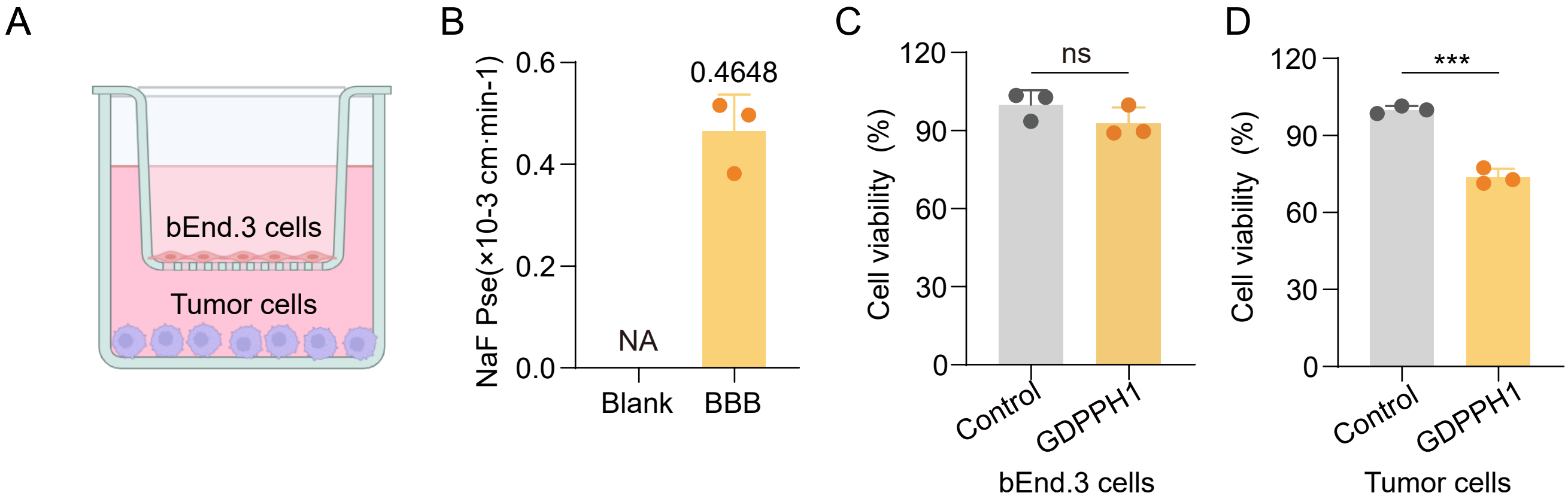
A
bEnd.3 cells
Tumor cells
B
NaF Pse(×10-3 cm·min-1)
0.6
0.4
0.2
0.0
0.4648
NA
Blank
BBB
C
120
90
60
30
0
ns
Cell viability (%)
Control
GDPPH1
bEnd.3 cells
D
120
90
60
30
0
***
Cell viability (%)
Control
GDPPH1
Tumor cells
