# Supplementary material for: GDPPH1 induces cell cycle arrest by downregulation of CDK4/Cyclin D1 via the m6A-METTL14-YTHDF2 axis, attenuating glioblastoma progression
Source: Cell Death Dis. 2026 Jul 3;17(1):828. doi: 10.1038/s41419-026-09036-x (PMC13601516; doi:10.1038/s41419-026-09036-x)
Supplement: Supplementary file 6 [file 41419_2026_9036_MOESM1_ESM.pdf]

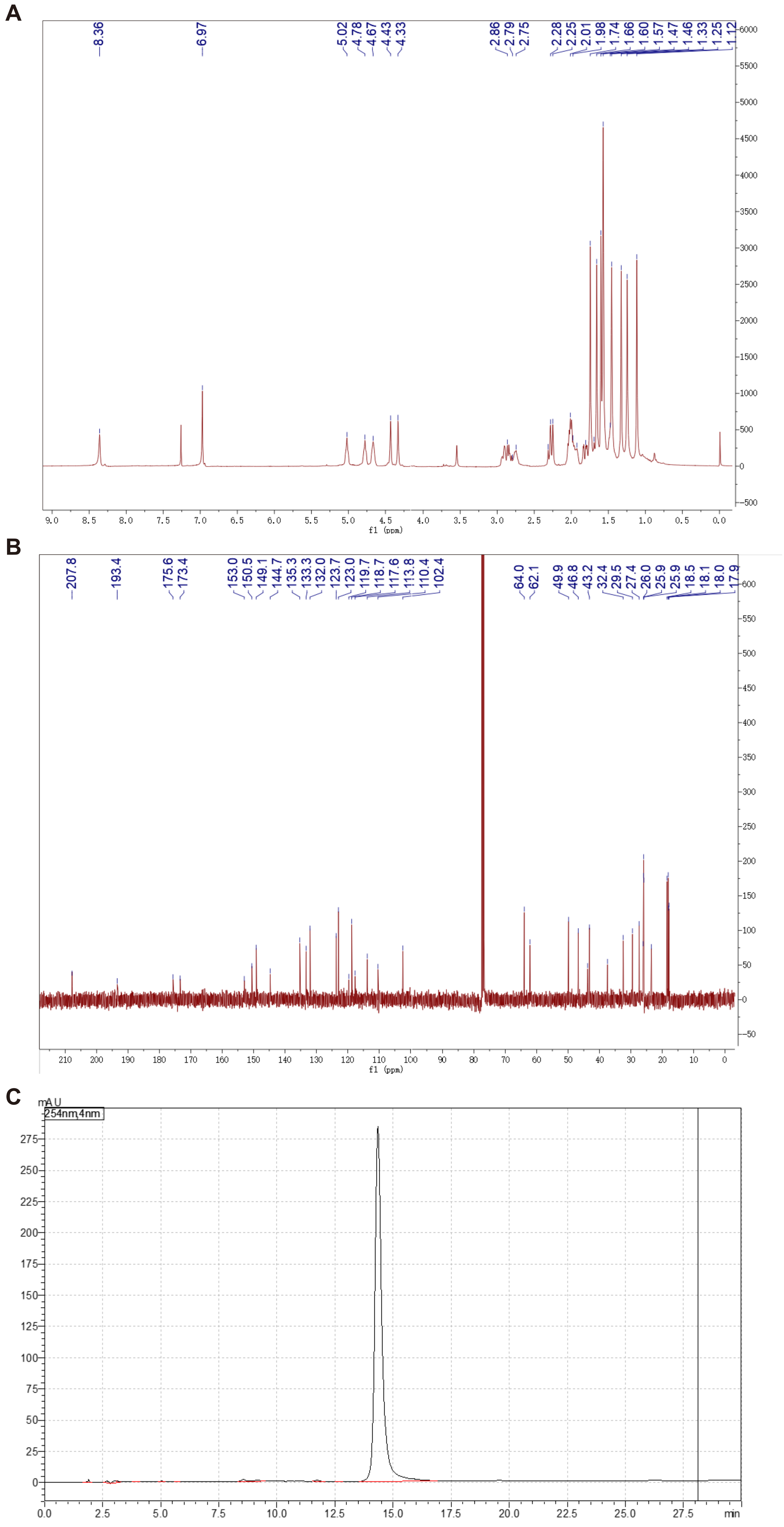

A
8.36
6.97
5.02
4.78
4.67
4.43
4.33
2.86
2.79
2.75
2.28
2.25
2.01
1.98
1.74
1.66
1.60
1.57
1.47
1.46
1.33
1.25
1.12
f1 (ppm)
B
207.8
193.4
175.6
173.4
153.0
150.5
149.1
144.7
135.3
133.3
132.0
123.7
123.0
119.7
118.7
117.6
113.8
110.4
102.4
64.0
62.1
49.9
46.8
43.2
32.4
29.5
27.4
26.0
25.9
25.9
18.5
18.1
18.0
17.9
f1 (ppm)
C
mAU
254nm,4nm
min
