# Supplementary material for: GDPPH1 induces cell cycle arrest by downregulation of CDK4/Cyclin D1 via the m6A-METTL14-YTHDF2 axis, attenuating glioblastoma progression
Source: Cell Death Dis. 2026 Jul 3;17(1):828. doi: 10.1038/s41419-026-09036-x (PMC13601516; doi:10.1038/s41419-026-09036-x)
Supplement: Supplementary file 7 [file 41419_2026_9036_MOESM2_ESM.pdf]

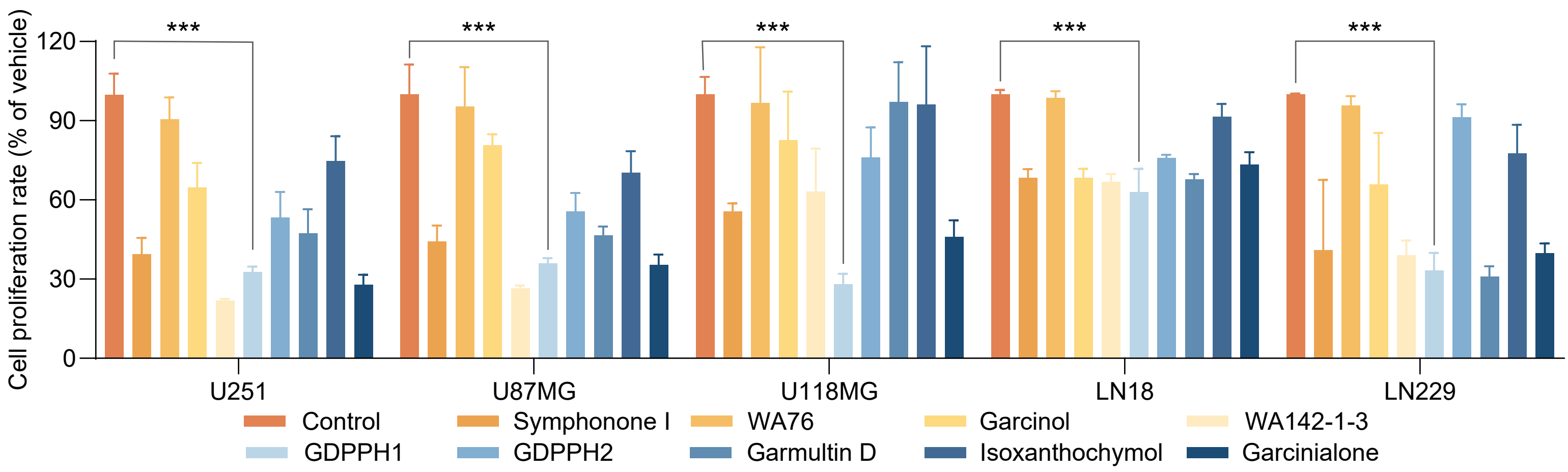
Cell proliferation rate (% of vehicle)
120
90
60
30
0
***
***
***
***
***
U251
U87MG
U118MG
LN18
LN229
Control
Symphonone I
WA76
Garcinol
WA142-1-3
GDPPH1
GDPPH2
Garmultin D
Isoxanthochymol
Garcinialone
