# Supplementary material for: GDPPH1 induces cell cycle arrest by downregulation of CDK4/Cyclin D1 via the m6A-METTL14-YTHDF2 axis, attenuating glioblastoma progression
Source: Cell Death Dis. 2026 Jul 3;17(1):828. doi: 10.1038/s41419-026-09036-x (PMC13601516; doi:10.1038/s41419-026-09036-x)
Supplement: Supplementary file 8 [file 41419_2026_9036_MOESM3_ESM.pdf]

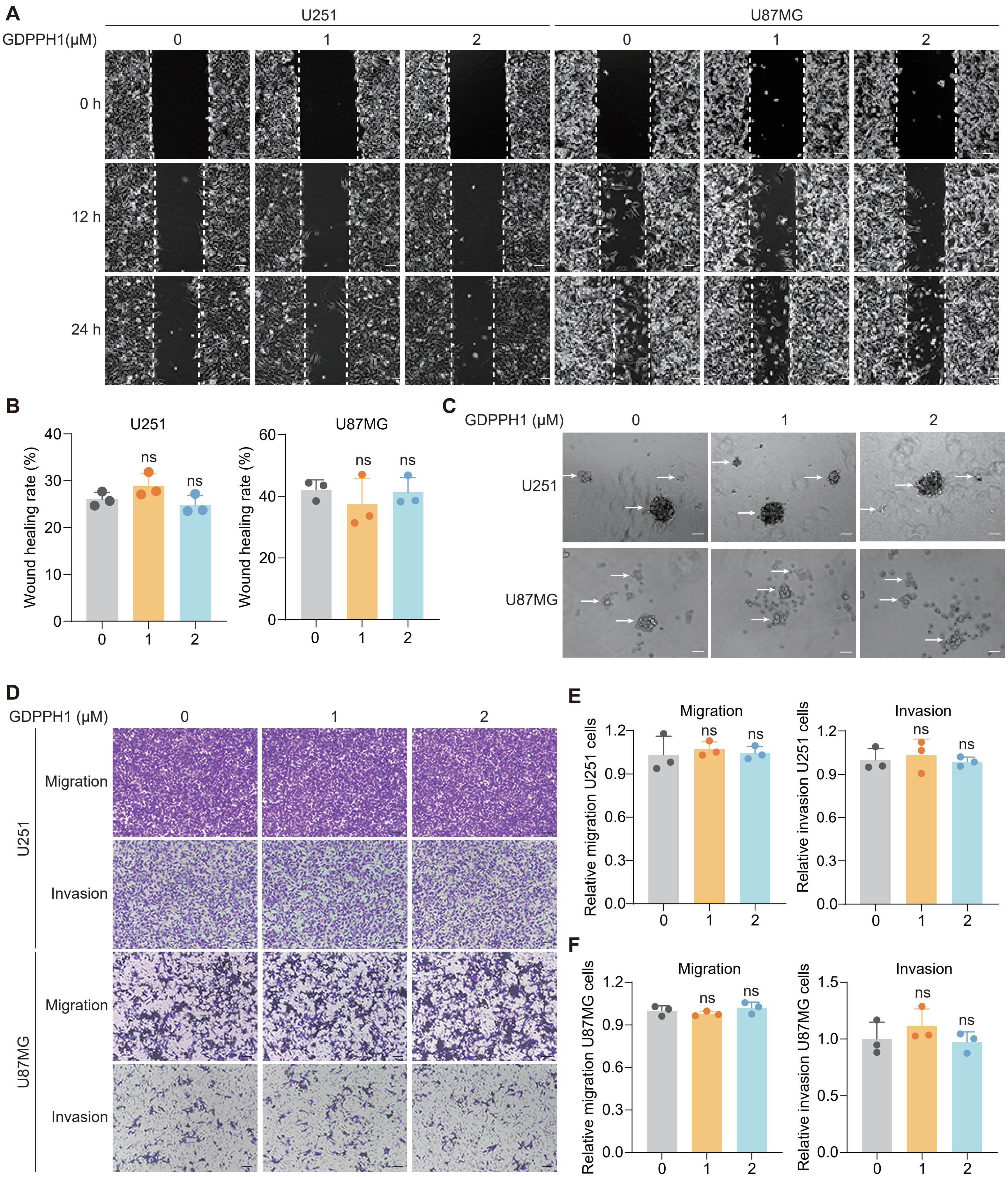

A
U251
U87MG
GDPPH1(μM)
0
1
2
0 h
12 h
24 h
B
U251
U87MG
Wound healing rate (%)
ns
C
GDPPH1 (μM)
D
GDPPH1 (μM)
Migration
Invasion
E
Relative migration U251 cells
Relative invasion U251 cells
F
Relative migration U87MG cells
Relative invasion U87MG cells
