# Supplementary material for: GDPPH1 induces cell cycle arrest by downregulation of CDK4/Cyclin D1 via the m6A-METTL14-YTHDF2 axis, attenuating glioblastoma progression
Source: Cell Death Dis. 2026 Jul 3;17(1):828. doi: 10.1038/s41419-026-09036-x (PMC13601516; doi:10.1038/s41419-026-09036-x)
Supplement: Supplementary file 9 [file 41419_2026_9036_MOESM4_ESM.pdf]

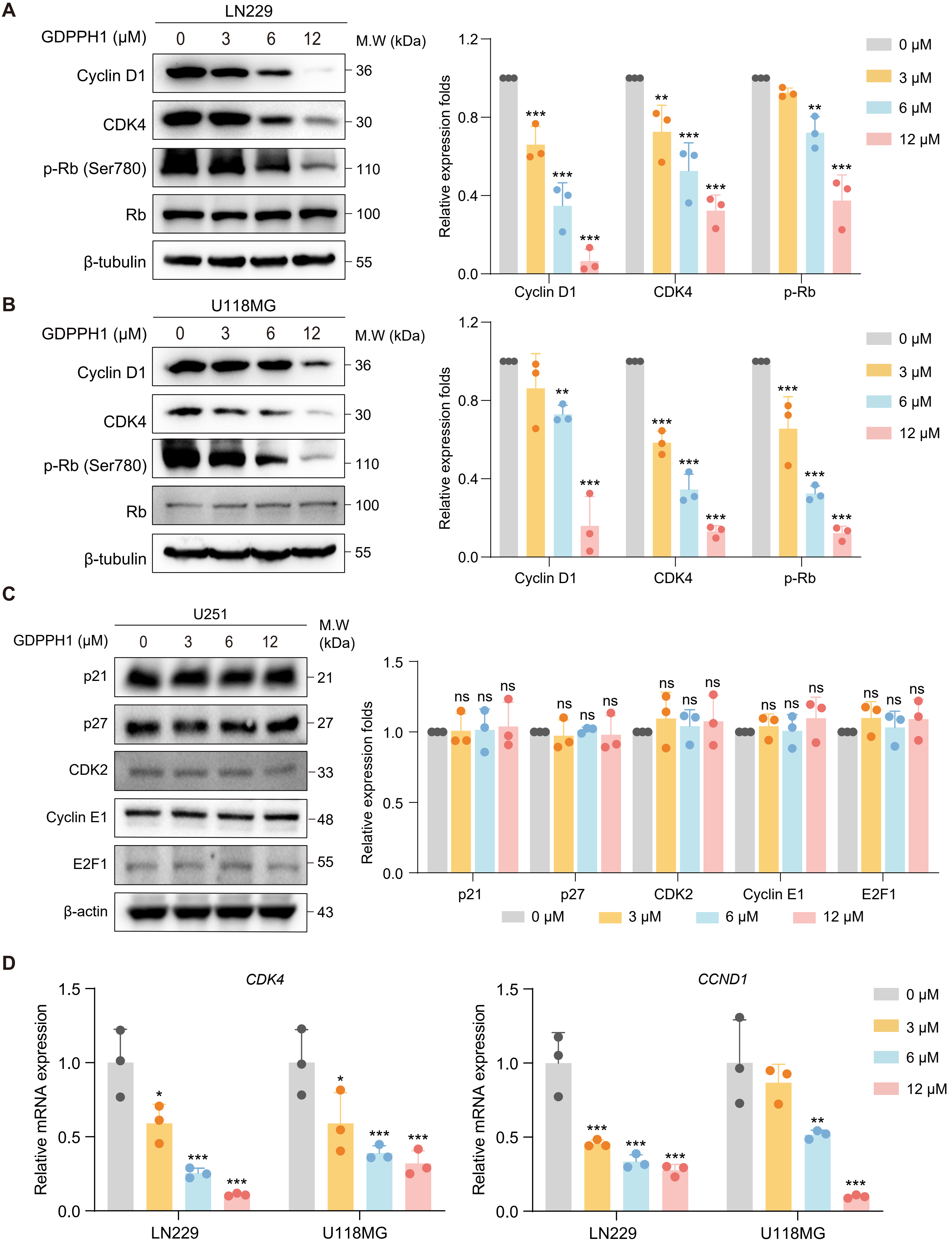
A
LN229
GDPPH1 (μM) 0 3 6 12
M.W (kDa)
Cyclin D1 36
CDK4 30
p-Rb (Ser780) 110
Rb 100
β-tubulin 55
Relative expression folds
0 μM
3 μM
6 μM
12 μM
Cyclin D1
CDK4
p-Rb
B
U118MG
GDPPH1 (μM) 0 3 6 12
M.W (kDa)
Cyclin D1 36
CDK4 30
p-Rb (Ser780) 110
Rb 100
β-tubulin 55
Relative expression folds
0 μM
3 μM
6 μM
12 μM
Cyclin D1
CDK4
p-Rb
C
U251
GDPPH1 (μM) 0 3 6 12
M.W (kDa)
p21 21
p27 27
CDK2 33
Cyclin E1 48
E2F1 55
β-actin 43
Relative expression folds
p21
p27
CDK2
Cyclin E1
E2F1
0 μM
3 μM
6 μM
12 μM
D
CDK4
CCND1
Relative mRNA expression
LN229
U118MG
0 μM
3 μM
6 μM
12 μM
