# Supplementary material for: GDPPH1 induces cell cycle arrest by downregulation of CDK4/Cyclin D1 via the m6A-METTL14-YTHDF2 axis, attenuating glioblastoma progression
Source: Cell Death Dis. 2026 Jul 3;17(1):828. doi: 10.1038/s41419-026-09036-x (PMC13601516; doi:10.1038/s41419-026-09036-x)
Supplement: Supplementary file 10 [file 41419_2026_9036_MOESM5_ESM.pdf]

A

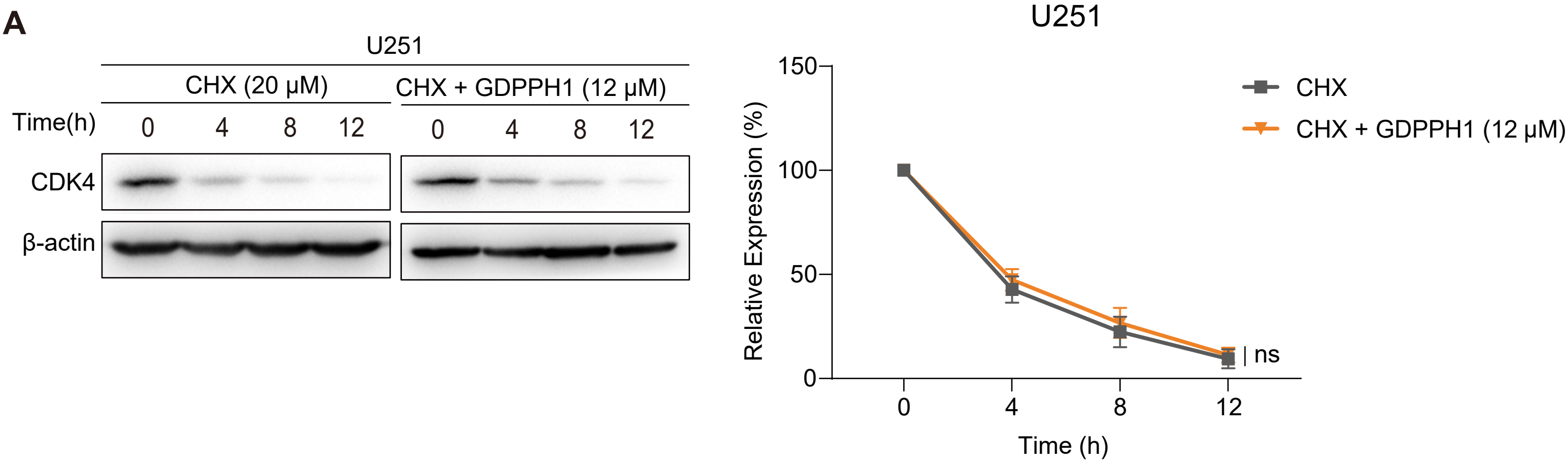

U251
CHX (20 μM)
CHX + GDPPH1 (12 μM)
Time(h)
0
4
8
12
CDK4
β-actin
U251
150
100
50
0
Relative Expression (%)
Time (h)
CHX
CHX + GDPPH1 (12 μM)
ns

B

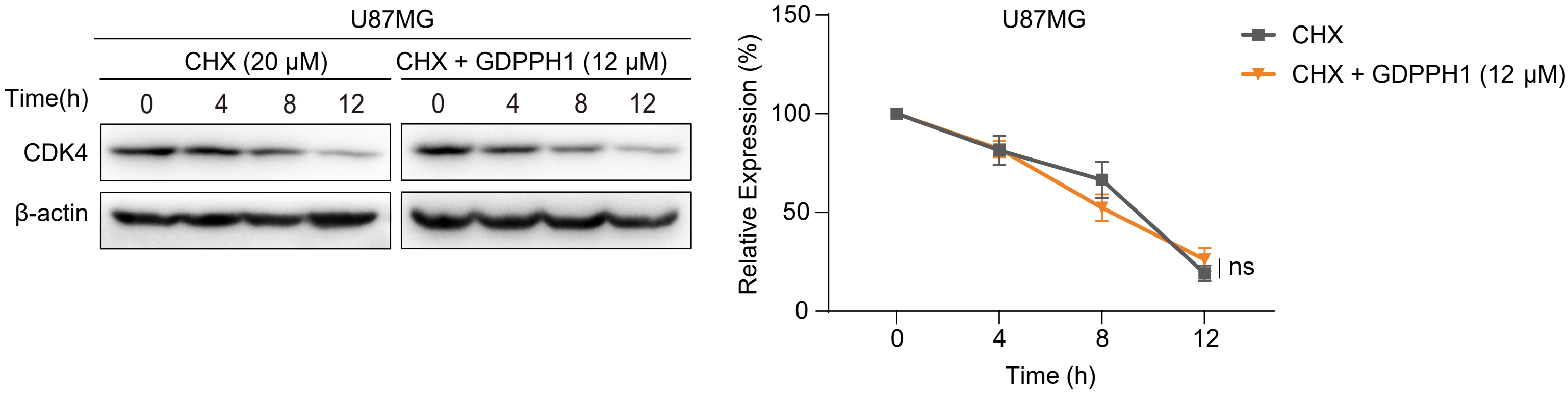

U87MG
CHX (20 μM)
CHX + GDPPH1 (12 μM)
Time(h)
0
4
8
12
CDK4
β-actin
U87MG
150
100
50
0
Relative Expression (%)
Time (h)
CHX
CHX + GDPPH1 (12 μM)
ns
