# Supplementary material for: GDPPH1 induces cell cycle arrest by downregulation of CDK4/Cyclin D1 via the m6A-METTL14-YTHDF2 axis, attenuating glioblastoma progression
Source: Cell Death Dis. 2026 Jul 3;17(1):828. doi: 10.1038/s41419-026-09036-x (PMC13601516; doi:10.1038/s41419-026-09036-x)
Supplement: Supplementary file 11 [file 41419_2026_9036_MOESM6_ESM.pdf]

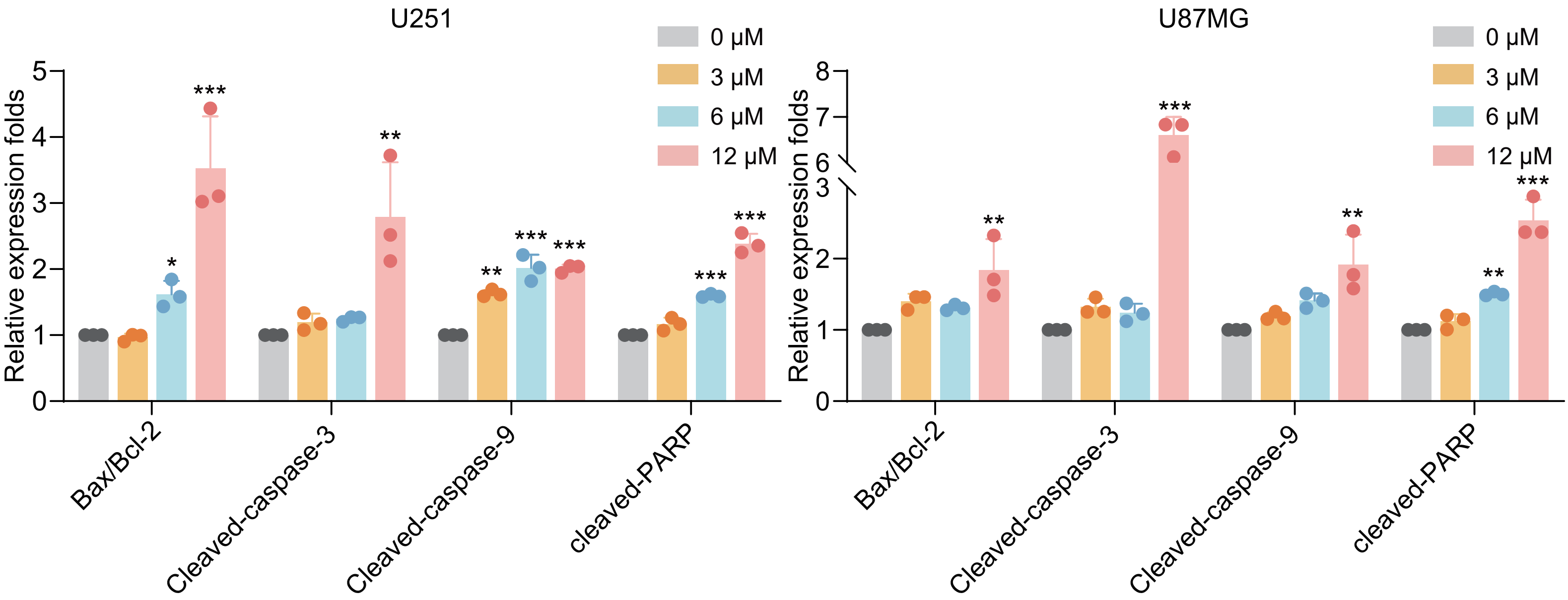
U251
U87MG
0 μM
3 μM
6 μM
12 μM
Relative expression folds
Bax/Bcl-2
Cleaved-caspase-3
Cleaved-caspase-9
cleaved-PARP
*
**
***
