# Supplementary material for: GDPPH1 induces cell cycle arrest by downregulation of CDK4/Cyclin D1 via the m6A-METTL14-YTHDF2 axis, attenuating glioblastoma progression
Source: Cell Death Dis. 2026 Jul 3;17(1):828. doi: 10.1038/s41419-026-09036-x (PMC13601516; doi:10.1038/s41419-026-09036-x)
Supplement: Supplementary file 12 [file 41419_2026_9036_MOESM7_ESM.pdf]

A

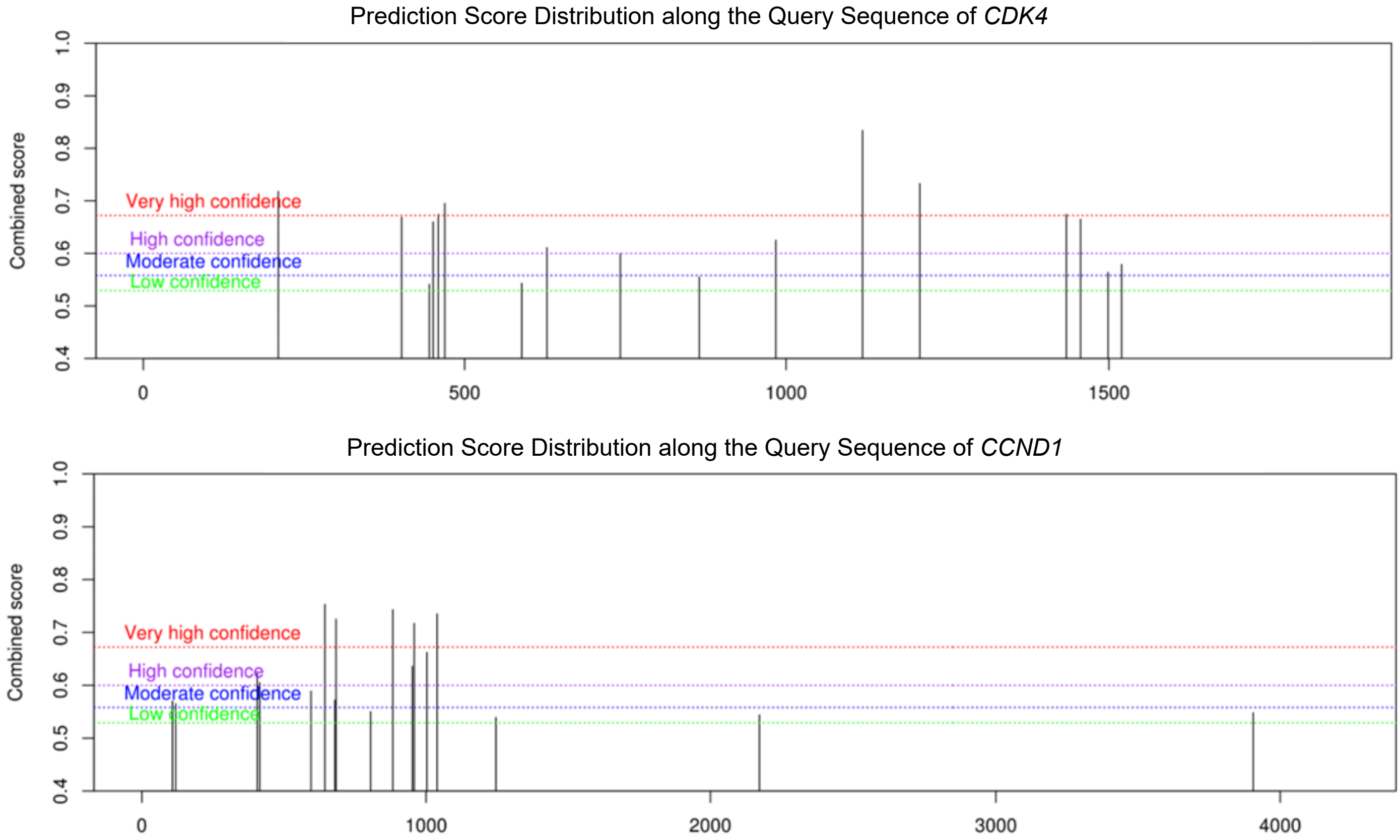
Prediction Score Distribution along the Query Sequence of CDK4
Combined score
Very high confidence
High confidence
Moderate confidence
Low confidence
Prediction Score Distribution along the Query Sequence of CCND1

B

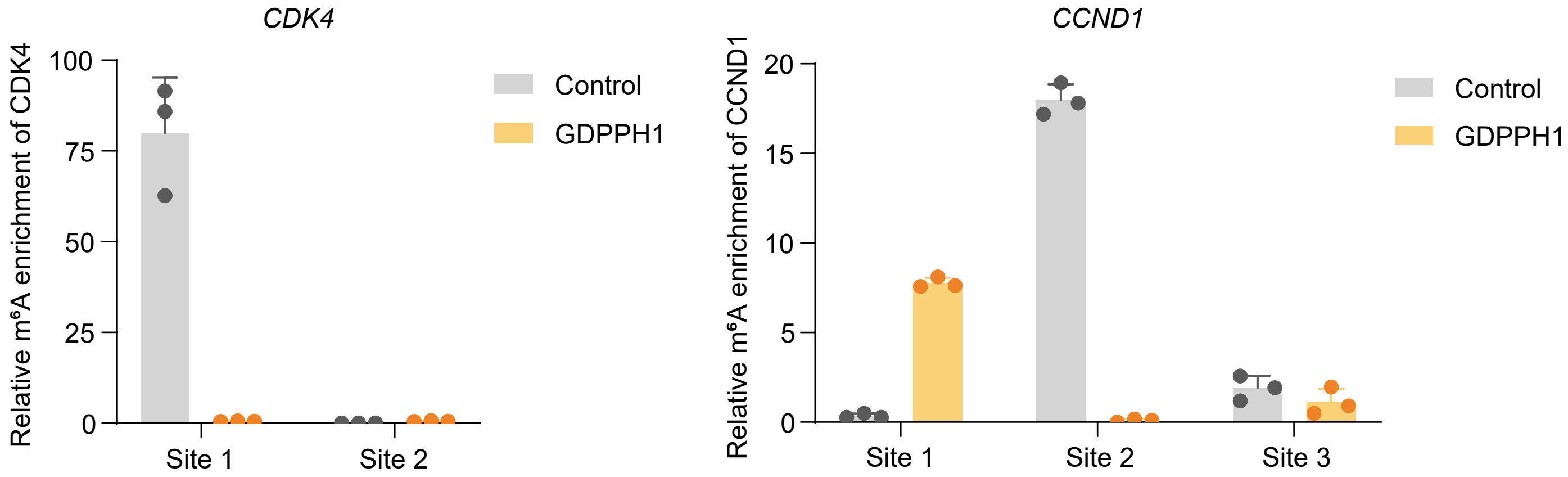
CDK4
Relative m6A enrichment of CDK4
Control
GDPPH1
Site 1
Site 2
CCND1
Relative m6A enrichment of CCND1
Site 3
