# Supplementary material for: GDPPH1 induces cell cycle arrest by downregulation of CDK4/Cyclin D1 via the m6A-METTL14-YTHDF2 axis, attenuating glioblastoma progression
Source: Cell Death Dis. 2026 Jul 3;17(1):828. doi: 10.1038/s41419-026-09036-x (PMC13601516; doi:10.1038/s41419-026-09036-x)
Supplement: Supplementary file 13 [file 41419_2026_9036_MOESM8_ESM.pdf]

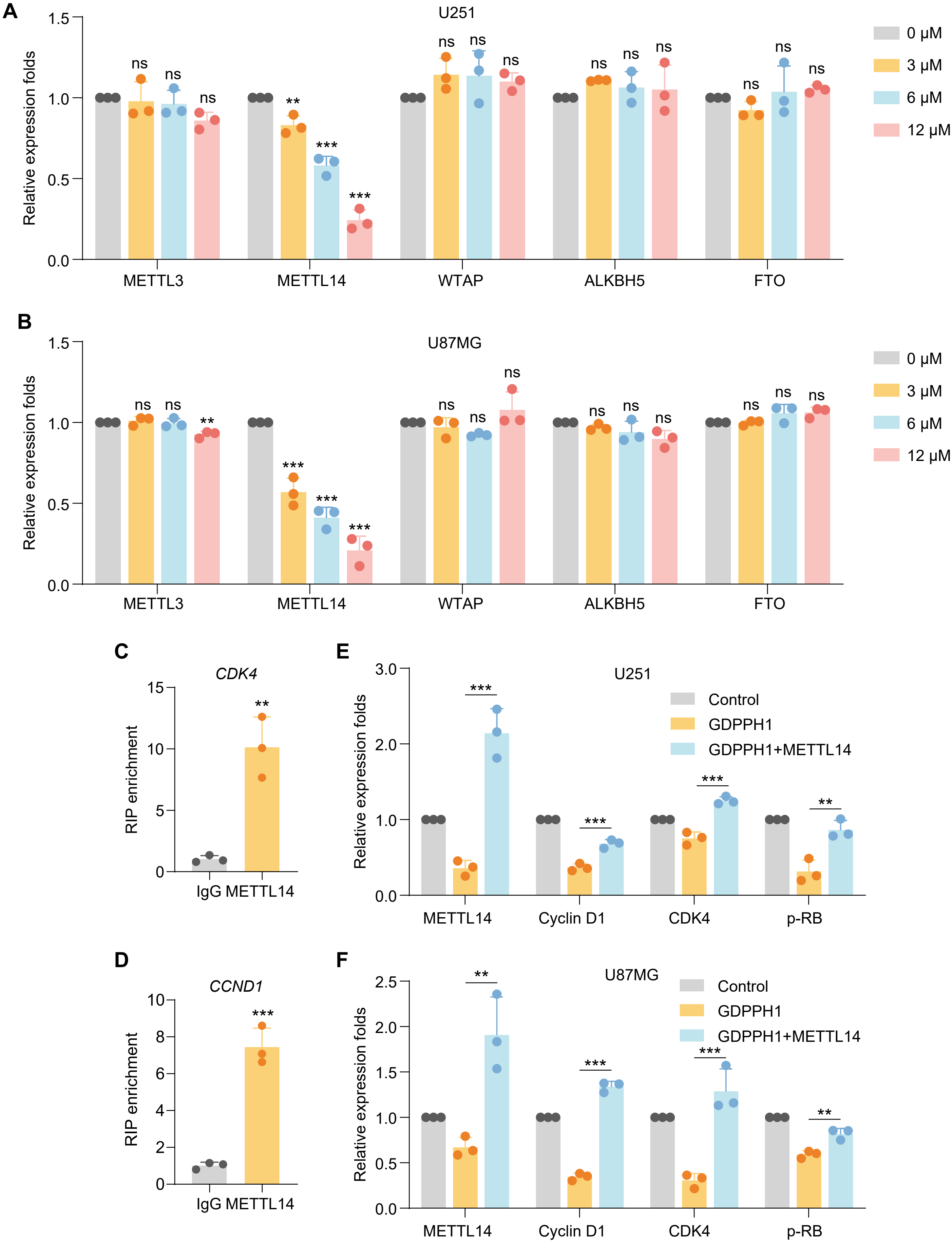
A
U251
Relative expression folds
0 μM
3 μM
6 μM
12 μM
METTL3
METTL14
WTAP
ALKBH5
FTO
B
U87MG
Relative expression folds
C
CDK4
RIP enrichment
IgG METTL14
D
CCND1
RIP enrichment
IgG METTL14
E
U251
Relative expression folds
Control
GDPPH1
GDPPH1+METTL14
METTL14
Cyclin D1
CDK4
p-RB
F
U87MG
Relative expression folds
Control
GDPPH1
GDPPH1+METTL14
METTL14
Cyclin D1
CDK4
p-RB
