# Supplementary material for: GDPPH1 induces cell cycle arrest by downregulation of CDK4/Cyclin D1 via the m6A-METTL14-YTHDF2 axis, attenuating glioblastoma progression
Source: Cell Death Dis. 2026 Jul 3;17(1):828. doi: 10.1038/s41419-026-09036-x (PMC13601516; doi:10.1038/s41419-026-09036-x)
Supplement: Supplementary file 14 [file 41419_2026_9036_MOESM9_ESM.pdf]

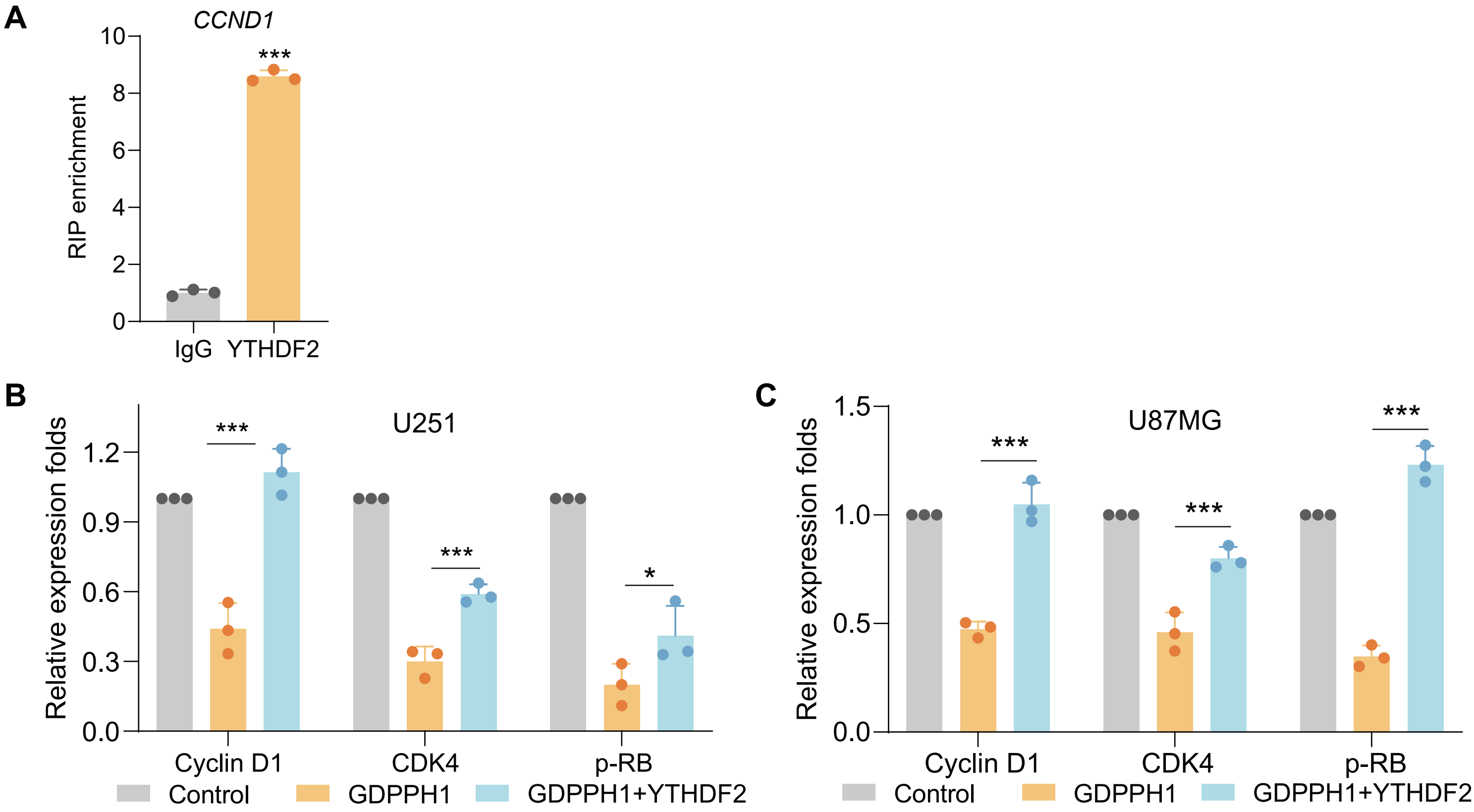
A
CCND1
RIP enrichment
10
8
6
4
2
0
***
IgG
YTHDF2
B
U251
Relative expression folds
1.2
0.9
0.6
0.3
0.0
***
***
*
Cyclin D1
CDK4
p-RB
Control
GDPPH1
GDPPH1+YTHDF2
C
U87MG
Relative expression folds
1.5
1.0
0.5
0.0
***
***
***
Cyclin D1
CDK4
p-RB
Control
GDPPH1
GDPPH1+YTHDF2
