# Supplementary material for: GDPPH1 induces cell cycle arrest by downregulation of CDK4/Cyclin D1 via the m6A-METTL14-YTHDF2 axis, attenuating glioblastoma progression
Source: Cell Death Dis. 2026 Jul 3;17(1):828. doi: 10.1038/s41419-026-09036-x (PMC13601516; doi:10.1038/s41419-026-09036-x)
Supplement: Supplementary file 15 [file 41419_2026_9036_MOESM14_ESM.docx]

**Supplementary Figure legends**

**Supplementary Figure 1 Purity and structural confirmation of GDPPH1**

(A) ^1^H NMR spectrum of GDPPH1 in CDCl_3_. (B) ^13^C NMR spectrum of GDPPH1 in CDCl_3_. (C) HPLC chromatogram of GDPPH1 purity >98%, calculated based on peak area.

**Supplementary Figure 2 Screening of candidate compounds isolated from mangosteen**

The cell viability was determined by MTT assay, after treatment with 9 candidate compounds (8 μM) isolated from mangosteen for 48 h. Data are presented as mean ± SD from at least three independent replicates. Statistical significance among multiple groups was performed using one-way ANOVA followed by Tukey’s post hoc test. *P* < 0.05 was considered statistically significant. **P* < 0.05, ***P* < 0.01, ****P* < 0.001.

**Supplementary Figure 3 GDPPH1 does not affect migration, invasion, or stemness in GBM cells**

(A) Representative images from wound healing assay of U251 and U87MG cells. (B) Quantification of the wound healing rate in A. (C) Representative images of tumor spheres in U251 and U87MG cells. (D) Effect of GDPPH1 on migration and invasion in U251 and U87MG cells. Following treatment with GDPPH1 (0, 1, or 2 μM) for 24 h, the cells were subjected to the Transwell migration and invasion assays. Scale bars, 100 μm. (E, F) Quantification of the migration and invasion rates in D. Data are presented as mean ± SD from at least three independent replicates. Statistical significance among multiple groups was performed using one-way ANOVA followed by Tukey’s post hoc test. *P* < 0.05 was considered statistically significant. **P* < 0.05, ***P* < 0.01, ****P* < 0.001.

**Supplementary Figure 4 GDPPH1 induced G1/S phase arrest through reducing the expression of cell cycle-associated proteins in glioblastoma cells**

(A, B) GDPPH1 (3, 6, and 12 μM) treatment for 12 h decreased the protein expression of Cyclin D1, CDK4, p-RB (Ser780), and RB in LN229 and U118MG cell lines. β-tubulin was used as the loading control. (C) Western blot analysis showing no significant changes in the expression levels of p21, p27, CDK2, Cyclin E1, and E2F1 in GBM cells after GDPPH1 treatment. (D) Relative mRNA expression of *CDK4* and *CCND1* in LN229 and U118MG cell lines with 12 h GDPPH1 treatment at 3, 6, and 12 μM. Data are presented as mean ± SD from at least three independent replicates. Statistical significance among multiple groups was performed using one-way ANOVA followed by Tukey’s post hoc test. *P* < 0.05 was considered statistically significant. **P* < 0.05, ***P* < 0.01, ****P* < 0.001.

**Supplementary Figure 5 GDPPH1 does not alter the stability of CDK4 and Cyclin D1**

(A, B) U251 and U87MG cells with or without GDPPH1 were treated with 5 μg/mL cycloheximide (CHX) for the indicated time, and the stability of CDK4 was determined by immunoblotting. β-actin was used as the loading control. Protein half-life was calculated using one-phase exponential decay analysis. *P* < 0.05 was considered statistically significant. **P* < 0.05, ***P* < 0.01, ****P* < 0.001.

**Supplementary Figure 6 Quantification of Western blot analysis of apoptosis-related proteins in GBM cells treated with GDPPH1**

Quantification of the expression of indicated protein levels in Fig. 3B. Data are presented as mean ± SD from at least three independent replicates. Statistical significance among multiple groups was performed using one-way ANOVA followed by Tukey’s post hoc test. *P* < 0.05 was considered statistically significant. **P* < 0.05, ***P* < 0.01, ****P* < 0.001.

**Supplementary Figure 7 Prediction and confirmation of potential m^6^A binding site on *CDK4* and *CCND1* mRNA**

(A) m^6^A modification sites on *CDK4* and *CCND1* mRNA predicted with SRAMP. (B) Confirmation of m⁶A enrichment of *CDK4* and *CCND1* on different m^6^A modification sites with very high confidence. Data are presented as mean ± SD from at least three independent replicates. Statistical significance between two groups was determined using unpaired two-tailed Student’s t-test, while comparisons among multiple groups were performed using one-way ANOVA followed by Tukey’s post hoc test. *P* < 0.05 was considered statistically significant. **P* < 0.05, ***P* < 0.01, ****P* < 0.001.

**Supplementary Figure 8 METTL14 regulates *CDK4* and *CCND1* mRNA expression and directly binds to their mRNA**

(A, B) Quantification of the expression of indicated protein levels in Fig. 5B. (C, D) Interaction between METTL14 and *CDK4*, *CCND1* detected by RIP. (E, F) Quantification of the expression of indicated protein levels in Fig. 5D. Data are presented as mean ± SD from at least three independent replicates. Statistical significance between two groups was determined using unpaired two-tailed Student’s t-test, while comparisons among multiple groups were performed using one-way ANOVA followed by Tukey’s post hoc test. *P* < 0.05 was considered statistically significant. **P* < 0.05, ***P* < 0.01, ****P* < 0.001.

**Supplementary Figure 9 YTHDF2 directly binds to *CCND1* mRNA and regulates *CDK4* and *CCND1* mRNA expression**

(A) Interaction between YTHDF2 and *CCND1* detected by RIP assay. (B, C) Quantification of the indicated protein expression levels shown in Fig. 6D.

**Supplementary Figure 10 GDPPH1 treatment did not induce significant changes in the activation or expression levels of several key oncogenic pathways**

(A) Western blotting analysis shows no significant changes in the expression of p-ERK1/2 (Thr202/Tyr204), ERK1/2, p-Akt (Ser473), Akt, p-AMPK (Thr172), AMPK, p-YAP (Ser127), YAP, p-β-Catenin (Ser675) or β-Catenin after GDPPH1 treatment. (B) Quantification of the expression of indicated protein levels in A. Data are presented as mean ± SD from at least three independent replicates. Statistical significance were performed using one-way ANOVA followed by Tukey’s post hoc test. *P* < 0.05 was considered statistically significant. **P* < 0.05, ***P* < 0.01, ****P* < 0.001.

**Supplementary Figure 11 Evaluation of the safety of GDPPH1 *in vivo***

(A) No significant changes in body weights were observed after GDPPH1 treatment. (B-H) Levels of ALT, AST, CK, CREA, WBC count, Hemoglobin, and Platelets in the blood of nude mice bearing transplanted tumors. Scale bar, 75 μm. (I) Representative H&E staining of liver, heart, spleen, lung, and kidney. Scale bar, 20 μm. Data are presented as mean ± SD from at least three independent replicates. Statistical significance among multiple groups were performed using one-way ANOVA followed by Tukey’s post hoc test. *P* < 0.05 was considered statistically significant. **P* < 0.05, ***P* < 0.01, ****P* < 0.001.

**Supplementary Figure 12 *CDK4*, *CCND1*, *METTL14*, and *YTHDF2* are highly expressed in GBM and are potentially involved in gliomagenesis**

(A-D) Comparison of *CDK4*, *CCND1*, *METTL14*, and *YTHDF2* expression levels between normal brain and GBM tissues within the TCGA dataset. (E) Analysis of *CDK4* expression levels across glioma tissues of WHO Grades II, III, and IV within the CGGA dataset. (F-I) Evaluation of *CDK4*, *CCND1*, *METTL14*, and *YTHDF2* expression levels across primary and recurrent GBM. Data are presented as mean ± SD from at least three independent replicates. Statistical significance between two groups was determined using unpaired two-tailed Student’s t-test, while comparisons among multiple groups were performed using one-way ANOVA followed by Tukey’s post hoc test. *P* < 0.05 was considered statistically significant. **P* < 0.05, ***P* < 0.01, ****P* < 0.001.

**Supplementary Figure 13 Evaluation of GDPPH1 permeability in an in vitro BBB model**

(A) Schematic representation of the BBB permeability assay using bEnd.3 monolayer cultured on a Transwell membrane. (B) Cell viability of bEend.3 cells measured by MTT assay after treatment with GDPPH1 (12 μM) for 48 h. (C) Cell viability of GBM cells determined by MTT assay after treatment with GDPPH1 for 48 h. (D) The data of sodium fluorescein permeability tests (Na-Flu) indicated that the BBB model *in vitro* formed a sufficiently tight barrier layer. Data are presented as mean ± SD from at least three independent replicates. Statistical significance between two groups was determined using an unpaired two-tailed Student’s t-test. *P* < 0.05 was considered statistically significant. **P* < 0.05, ***P* < 0.01, ****P* < 0.001.
