# Supplementary material for: GDPPH1 induces cell cycle arrest by downregulation of CDK4/Cyclin D1 via the m6A-METTL14-YTHDF2 axis, attenuating glioblastoma progression
Source: Cell Death Dis. 2026 Jul 3;17(1):828. doi: 10.1038/s41419-026-09036-x (PMC13601516; doi:10.1038/s41419-026-09036-x)
Supplement: Supplementary file 16 [file 41419_2026_9036_MOESM15_ESM.docx]

**Supplementary Table 1**

**Information of candidate compounds isolated from mangosteen**

| **Name** | **Molecular weight** | **Molecular structure** |
| --- | --- | --- |
| Symphonone I | 600.35 |  |
| WA76 | 550.69 |  |
| Garcinol (benzophenone) | 602.36 |  |
| WA142-1-3 | 600.35 |  |
| GDPPH1 (Garcim1) | 600.35 |  |
| GDPPH2 (Garcim2) | 600.35 |  |
| Garmultin D | 618.36 |  |
| Isoxanthochymol | 602.36 |  |
| Garcinialone | 618.36 |  |

**Supplementary Table 2**

**NMR Data of GDPPH1 (δ in parts per million, J in hertz)^a^**

| no. | *δ*_H_ | *δ*_C_ | |  | no. | *δ*_H_ | *δ*_C_ | |
| --- | --- | --- | --- | --- | --- | --- | --- | --- |
| 1 | - | 193.3 | C |  | 20 | 1.60 s | 26.0 | CH_3_ |
| 2 | - | 119.7 | C |  | 21 | 1.74 s | 18.5 | CH_3_ |
| 3 | - | 175.7 | C |  | 22 | 1.33 s | 23.4 | CH_3_ |
| 4 | - | 63.9 | C |  | 23 | 1.25 s | 27.4 | CH_3_ |
| 5 | - | 49.9 | C |  | 24 | 1.69 m, 1.92 m | 29.5 | CH_2_ |
| 6 | 1.47 m | 46.8 | CH |  | 25 | 4.67 d (7.5) | 123.7 | CH |
| 7 | 2.25 m, 2.28 m | 43.8 | CH_2_ |  | 26 | - | 133.3 | C |
| 8 | - | 62.2 | C |  | 27 | 1.46 s | 25.9 | CH_3_ |
| 9 | - | 207.8 | C |  | 28 | 1.12 s | 17.8 | CH_3_ |
| 10 | - | 173.4 | C |  | 29 | 1.81 m, 2.31 overlap | 37.4 | CH_2_ |
| 11 |  | 117.6 | C |  | 30 | 2.75 m | 43.2 | CH |
| 12 | 8.36 s | 110.1 | CH |  | 31 | - | 149.1 | C |
| 13 | - | 150.5 | C |  | 32 | 4.33 s, 4.43 s | 113.8 | CH_2_ |
| 14 | - | 153.0 | C |  | 33 | 1.57 s | 18.1 | CH_3_ |
| 15 | 6.97 s | 102.4 | CH |  | 34 | 1.98 m, 2.01 m | 32.4 | CH_2_ |
| 16 | - | 144.7 | C |  | 35 | 5.02 t (6.8) | 122.9 | CH |
| 17 | 2.79 m, 2.86 m | 26.1 | CH_2_ |  | 36 | - | 132.0 | C |
| 18 | 4.78 t (6.8) | 118.7 | CH |  | 37 | 1.66 s | 25.9 | CH_3_ |
| 19 | - | 135.3 | C |  | 38 | 1.57 s | 18.1 | CH_3_ |
| ^a^ Measured at 400 (^1^H) and 100 (^13^C) MHz in CDCl3. | | | | | | | | |

**Supplementary Table 3**

**Primer sequences for Real-time PCR**

| Gene | Species | Forward (5' -> 3') | Reverse (5' -> 3') |
| --- | --- | --- | --- |
| CDK4 | human | CTGGTGTTTGAGCATGTAGACC | GATCCTTGATCGTTT CGGCTG |
| CCND1 | human | CCGAGAAGCTGTGCATCTACACCG | CTGTTTGTTCTCCTCCGCCTCTGG |
| METTL3 | human | ATCCCCAAGGCTTCAACCAG | GCGAGTGCCAGGAGATAGTC |
| METTL14 | human | AGAGAACAAAGGAACACTGCCT | AATGAAGTCCCCGTCTGTGC |
| WTAP | human | GCGGTGGCAGTGTACAGTTA | CCTGCCTTCGAGATGAGAGT |
| ALKBH5 | human | TGACTGTGCTCAGTGGATATG | TGACAGGCGATCTGAAGCAT |
| FTO | human | GCGGTGGCAGTGTACAGTTA | CCTGCCTTCGAGATGAGAGT |
| YTHDF1 | human | CGTGGACACCCAGAGAACAA | TAGCTGGACAGGTAGGGGTC |
| YTHDF2 | human | GTCAGGGACAAAAGCCTCCG | GACCTTTTGGTCTCTGCTCCA |
| YTHDF3 | human | GGTGTATTTAGTCAACCTGGGG | AAGAGAACTAGGTGGATAGCCAT |
| GAPDH | human | GACTCATGACCACAGTCCATGC | AGAGGCAGGGATGATGTTCTG |

**Supplementary Table 4**

**Primer sequences for MeRIP-RT-qPCR**

| Gene | Position | Species | Forward (5' -> 3') | Reverse (5' -> 3') |
| --- | --- | --- | --- | --- |
| CDK4 | 1 | human | TGGCTGCCATGGAAGGAAGAAAA | AGGACCCTCCATAGCCTCAGA |
|  | 2 | human | GGAGGGTCCTCCTCCATCTTT | ATGGGAAGGAGAAGGAGAAGCC |
| CCND1 | 1 | human | GCAATGACCCCGCACGAT | CCGCCTCTGGCATTTTGG |
|  | 2 | human | CACGCTTCCTCTCCAGAGTGA | CTTCGATCTGCTCCTGGCAG |
|  | 3 | human | TTGCACACCCACCGACGT | TGGCGCCCGCCTG |

**Supplementary Table 5**

**Average GBM inhibition rate of candidate compounds**

| **Drug** | **Average inhibition rate (mean ± SD)** |
| --- | --- |
| Symphonone I | 49.48 ± 14.26% |
| WA76 | 6.42 ± 10.53% |
| Garcinol (benzophenone) | 27.84 ± 11.94% |
| WA142-1-3 | 56.50 ± 19.68% |
| **GDPPH1 (Garcim1)** | **60.83 ± 13.24%** |
| GDPPH2 (Garcim2) | 29.97 ± 15.18% |
| Garmultin D | 42.56 ± 20.40% |
| Isoxanthochymol | 18.11 ± 13.41% |
| Garcinialone | 55.36 ± 17.18% |
